# Synthesis of new imine-/amine-bearing imidazo[1,2-a]pyrimidine derivatives and screening of their cytotoxic activity

**DOI:** 10.55730/1300-0527.3594

**Published:** 2023-10-11

**Authors:** Tuğba GÜNGÖR, Hazal Nazlıcan ATALAY, Yakup Berkay YILMAZ, Tuğba BOYUNEĞMEZ TÜMER, Mehmet AY

**Affiliations:** 1 Department of Chemistry, Faculty of Sciences, Çanakkale Onsekiz Mart University, Çanakkale, Turkiye; 2 Graduate Program of Molecular Biology and Genetics, School of Graduate Studies, Çanakkale Onsekiz Mart University, Çanakkale, Turkiye; 3 Department of Molecular Biology and Genetics, Faculty of Sciences, Çanakkale Onsekiz Mart University, Çanakkale, Turkiye

**Keywords:** Breast cancer, cytotoxicity, imidazo[1, 2-*a*]pyrimidine, imine, microwave

## Abstract

Imidazo[1,2-*a*]pyrimidine derivatives bearing imine groups (**3a**–**e**) were successfully synthesized in moderate to good yields using microwave-assisted heating. Corresponding amine derivatives (**4a**–**e**) were also obtained by the reduction reaction of the imine derivatives (**3a**–**e**). All synthesized products were characterized by FT-IR, ^1^H NMR, ^13^C NMR, and LC-MS spectroscopic techniques. In silico ADMET, Lipinski, and drug-likeness studies of the compounds were conducted and all were found to be suitable drug candidates. The cytotoxicity of the potential drug molecules was screened against the breast cancer cell lines MCF-7 and MDA–MB-231 and the healthy model HUVEC by the sulforhodamine B method. According to the antiproliferative studies, compounds **3d** and **4d** showed remarkable inhibition of MCF-7 cells with IC_50_ values of 43.4 and 39.0 μM and of MDA–MB-231 cells with IC_50_ values of 35.9 and 35.1 μM, respectively. In particular, compound **3d** selectively inhibited the proliferation of MCF-7 1.6-fold and MDA–MB-231 2.0-fold relative to healthy cells. Moreover, the apoptotic mechanism studies indicated that compound **4d** induced apoptosis by moderately increasing the ratio of Bax/Bcl-2 genes. Imidazo[1,2-*a*]pyrimidine derivative **3d**, a promising cytotoxic agent, may be helpful in the discovery of new and more efficient anticancer agents for breast cancer treatment.

## 1. Introduction

The imidazo[1,2-*a*]pyrimidine skeleton, which has an important position among nitrogen-containing heterocyclic scaffolds, has a broad range of medicinal applications such as anticancer, antimicrobial, antiviral, and antiinflammatory [1–6]. Divaplon, fasiplon, and taniplon, which are used as anxiolytic and anticonvulsant agents, are examples of imidazo[1,2-*a*]pyrimidine unit-containing drugs [7,8]. Furthermore, imines (Schiff bases) are well-known stable organic structures and numerous aromatic/heterocyclic molecules containing imine functional groups have various applications including in material science, chemistry, and biochemistry, especially pharmaceutical areas such as anticancer, antibacterial, antifungal, and antiinflammatory. [9–13]. Over the past years, our research team has reported some imidazo[1,2-*a*]pyrimidine compounds [14–16]. Therefore, combining imine and imidazo[1,2-*a*]pyrimidine cores in one molecule can be an interesting starting point from the synthesis and biological application perspectives.

Microwave-assisted organic synthesis has been an interesting field of study recently for increasing the reaction efficiency/selectivity/yield, shortening the reaction times, diminishing the decomposition/by-products, and therefore improving product purity [17–20]. This green method is extensively used for the synthesis of various heterocyclic compounds and imine derivatives in the literature [21–23]. Some imine-containing imidazo[1,2-*a*]pyrimidine derivatives were synthesized under microwave heating conditions by our research team [14].

**Figure 1:**
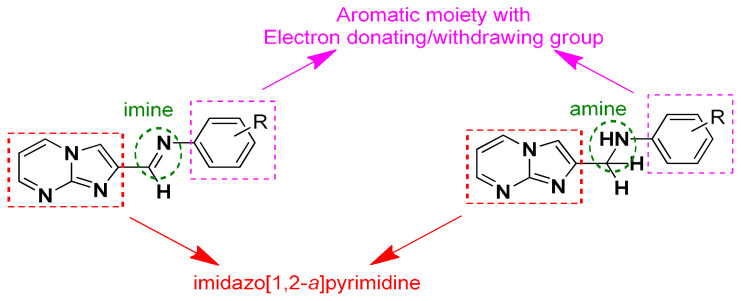
The general structure of imine-/amine-bearing imidazo[1,2-*a*]pyrimidine derivatives (**3a**–**e** and **4a**–**e**).

Breast cancer constitutes 25% of all cancer types seen in women worldwide [24,25]. Numerous FDA-approved drugs containing nitrogenous heterocyclic scaffolds in their structure, such as abemaciclib, palbociclib, olaparib, vinblastine, letrozole, and methotrexate are used for the treatment of breast cancer [26,27]. The goal of the present study was to investigate the synthesis of imidazo[1,2-*a*]pyrimidine derivatives bearing imine and amine and the screening of their cytotoxic activity against selected breast cancer cells. The first step was Schiff base formation (**3a**–**e)** between imidazo[1,2-*a*]pyrimidine-2-carbaldehyde and amine derivatives applying the microwave technique, which is efficient, simple, and ecofriendly. In the following step, imine derivatives were converted to the corresponding amines (**4a**–**e)** by reduction reaction (Figure 1). In the biological part of the investigation, the cytotoxic properties of the synthesized compounds were evaluated against the MDA–MB-231 and MCF-7 breast cancer cell lines and HUVEC healthy cells. Bax/Bcl-2 gene expression levels were examined in the MDA–MB-231 cell line by quantitative polymerase chain reaction (qPCR). Genes such as Bax and Bcl-2 play a vital role in intrinsic apoptosis as they control the fate of cells by promoting or inhibiting cell death. Bax acts as a promoter and Bcl-2 acts as an inhibitor of the apoptotic process. Imbalances in the expression or function of these genes can have significant implications for the detection of apoptosis [28]. Furthermore, in silico ADMET and drug-likeness studies of products were performed to identify drug feasibility.

## 2. Materials and methods

All chemicals were used without further purification. A CEM SP Discover Microwave Synthesizer was used to carry out the microwave-assisted synthesis in sealed vessels. X-4 and Electrothermal IA9100 melting point apparatuses were used to determine the melting points with a capillary tube. Alugram Xtra Sil G/UV_254_ plates were used for thin layer chromatography and visualized with UV irradiation for the monitoring of reaction progress. Products were purified by column chromatography on silica gel (Silicycle). A PerkinElmer Spectrum 100 FT-IR spectrometer with an ATR unit was used to record the FT-IR spectra in the region of 600–4000 cm^−1^. Jeol 400 MHz and Bruker Avance Neo 500 MHz NMR spectrometers were used to record the ^1^H and ^13^C NMR spectra of products in DMSO-d_6_. The residual solvent chemical shift signals are δ_H_ = 2.48 ppm and δ_C_ = 40.0 ppm for DMSO-d_6_ and δ_H_ = 3.33 ppm for HDO. A Shimadzu LC–MS/MS 8040 liquid chromatography mass spectrometer was used to record the mass spectra with electrospray ionization. The purities of the compounds were determined according to NMR analysis.

The main starting material, imidazo[1,2-*a*]pyrimidine-2-carbaldehyde, was synthesized according to the literature procedure [29]. Compounds **3a** (phenyl derivative) and **3b** (naphthyl derivative) were synthesized according to the reported procedure [14].

### 2.1. Synthesis of compounds 3c–e and 4a–e

#### General synthesis method for imine derivatives (3c–e)

A solution of imidazo[1,2-*a*]pyrimidine-2-carbaldehyde (1.0 equiv), amine derivatives (1.05 equiv), and MgSO_4_ (1.0 equiv) in ethyl alcohol was stirred at room temperature for 5 min in a 35-mL microwave vessel. The mixture was irradiated with the CEM Microwave system at 200 W power at 80–85 °C for an appropriate time (40–120 min). The reaction was completed by monitoring with TLC analysis and the solvent was evaporated under reduced pressure. Dichloromethane was added to the medium and then MgSO_4_ was filtered and washed with distilled water twice. After drying with Na_2_SO_4_, DCM was evaporated and purified by flash chromatography (hexane:ethyl acetate) to afford the desired imine compounds **3c**–**e**.

##### *N*-(4-Methoxyphenyl)-1-(imidazo[1,2-*a*]pyrimidin-2-yl)methanimine (3c)

The product was obtained using p-anisidine with 90 min reaction time. Yellow solid; 85%; mp 191–192 °C; IR (ATR) ϑ 3093, 3071, 3017, 2957, 1626, 1609, 1594, 1523, 1500, 1402, 1287, 1243, 1169, 1030, 834, 799, 777 cm^−1^; ^1^H NMR (400 MHz, DMSO-d_6_) δ 10.07 (dd, *J* = 6.8 and 1.9 Hz, 1H), 8.89 (s, 1H), 8.74 (dd, *J* = 4.2 and 1.9 Hz, 1H), 8.33 (s, 1H), 7.37–7.32 (m, *J* = 8.9 and 4.2 Hz, 3H), 6.98 (d, *J* = 8.9 Hz, 2H), 3.76 (s, 3H); ^13^C NMR (100 MHz, DMSO-d_6_) δ 158.43, 152.91, 150.76, 147.78, 144.37, 143.25, 137.18, 122.87, 121.43, 115.03, 111.33, 55.86; LC/MS (m/z): C_14_H_12_N_4_O requires 252.27 found [M + H]^+^: 253.0.

##### *N*-(4-(*N*^ı^*,N*^ı^-Diethylamino)phenyl)-1-(imidazo[1,2-*a*]pyrimidin-2-yl)methanimine (3d)

The product was obtained using N,N-diethyl-p-phenylenediamine with 40-min reaction time. Flash silica gel chromatography was used to purify the crude product with the hexane:ethyl acetate (1:2) solvent system. Yellow solid; 60%; mp 182–183 °C; IR (ATR) ϑ 3088, 3067, 2966, 2930, 1623, 1607, 1586, 1506, 1480, 1352, 1266, 1197, 1154, 1077, 1015, 866, 819, 798, 775, 721 cm^−1^; ^1^H NMR (400 MHz, DMSO-d_6_) δ 10.09 (dd, *J* = 6.8 and 2.1 Hz, 1H), 8.87 (s, 1H), 8.70 (dd, *J* = 4.2 and 2.1 Hz, 1H), 8.26 (s, 1H), 7.32–7.28 (d (2H) and dd (1H), *J* = 9.1 and 4.2 Hz), 6.68 (d, *J* = 9.1 Hz, 2H), 3.34 (q, *J* = 7.0 Hz, 4H), 1.07 (t, *J* = 7.0 Hz, 6H); ^13^C NMR (100 MHz, DMSO-d_6_) δ 152.42, 150.45, 146.98, 144.12, 142.09, 139.02, 136.98, 123.06, 121.78, 112.35, 111.08, 44.35, 13.04; LC/MS (m/z): C_17_H_19_N_5_ requires 293.37 found [M + H]^+^: 294.0.

##### *N*-(4-Trifluoromethylphenyl)-1-(imidazo[1,2-*a*]pyrimidin-2-yl)methanimine (3e)

The product was obtained using 4-(trifluoromethyl)aniline with 120-min reaction time. Flash silica gel chromatography was used to purify the crude product with the hexane:ethyl acetate (1:4) solvent system. Cream solid; 65%; mp 224–226 °C. IR (ATR) ϑ 3128, 3075, 3044, 1626, 1590, 1527, 1508, 1485, 1408, 1319, 1285, 1233, 1153, 1103, 1063, 1011, 964, 876, 796, 776, 739, 662 cm^−1^; ^1^H NMR (400 MHz, DMSO-d_6_) δ 10.02 (dd, *J* = 6.7 and 1.6 Hz, 1H), 8.92 (s, 1H), 8.79 (dd, *J* = 4.2 and 1.6 Hz, 1H), 8.44 (s, 1H), 7.77 (d, *J* = 8.1 Hz, 2H), 7.50 (d, *J* = 8.1 Hz, 2H), 7.39 (dd, *J* = 6.7 and 4.2 Hz, 1H); ^13^C NMR (125 MHz, DMSO-d_6_) δ 155.14, 153.57, 151.88, 151.22, 145.02, 137.38, 126.94 (q, *J_C-F_* = 3.5 Hz, 126,98, 126.95, 126.92, 126.90), 126.40 (q, *J_C-F_* = 32.0 Hz, 126.77, 126.52, 126.27, 126.02), 124.92 (q, *J_C-F_* = 270.0 Hz, 128.16, 126.00, 123.84, 121.69), 122.29, 121.08, 111.68; LC/MS (m/z): C_14_H_9_F_3_N_4_ requires 290.24 found [M + H]^+^: 291.0.

#### General synthesis method for amine derivatives (4a–e)

The imine derivative (1.0 equiv) was dissolved in methyl alcohol at room temperature. NaBH_4_ (4.0 equiv) was added over 30 min in small portions while the reaction medium was cooled in an ice bath. After the mixture was stirred at room temperature for 3–24 h, the solvent was evaporated to dryness under reduced pressure. Distilled water was added to the residue and extracted with DCM (3 times). The organic phase was washed with brine and water and dried with Na_2_SO_4_. The crude product obtained by solvent evaporation under reduced pressure was purified by column chromatography (ethyl acetate:methyl alcohol).

##### *N*-(Phenyl)-1-(imidazo[1,2-*a*]pyrimidin-2-yl)methanamine (4a)

Flash silica gel chromatography was used to purify the crude product with the EtOAc:MeOH (3:1) solvent system. Yellow solid; 70%; mp 194–197 °C; IR (ATR) ϑ 3341, 3109, 3066, 3011, 2972, 2899, 1615, 1602, 1511, 1497, 1439, 1321, 1300, 1271, 1178, 1145, 1057, 874, 793, 748, 696 cm^−1^; ^1^H NMR (400 MHz, DMSO-d_6_) δ 8.88 (dd, *J* = 6.9 and 2.0 Hz, 1H), 8.51 (dd, *J* = 4.0 and 1.9 Hz, 1H), 7.73 (s, 1H), 7.10–7.03 (dd (1H) and d (2H), *J* = 7.5 and 4.1 Hz), 6.66 (d, *J* = 7.8 Hz, 2H), 6.53 (t, *J* = 7.5 Hz, 1H), 6.13 (t, *J* = 5.4 Hz, 1H), 4.58 (d, *J* = 5.6 Hz, 2H); ^13^C NMR (100 MHz, DMSO-d_6_) δ 149.84, 148.60, 148.48, 133.96, 129.31, 121.30, 116.75, 112.92, 108.73, 36.96; LC/MS (m/z): C_13_H_12_N_4_ requires 224.27 found [M + H]^+^: 225.0.

##### *N*-(Naphthalen-1-yl)-1-(imidazo[1,2-*a*]pyrimidin-2-yl)methanamine (4b)

Flash silica gel chromatography was used to purify the crude product with the EtOAc:MeOH (10:1) solvent system. Yellow solid; 45%; mp 217–219 °C; IR (ATR) ϑ 3368, 3113, 3093, 3069, 3049, 3015, 2966, 2893, 1619, 1578, 1518, 1483, 1410, 1340, 1271, 1142, 1114, 1033, 866, 787, 772 cm^−1^; ^1^H NMR (400 MHz, DMSO-d_6_) δ 9.05 (dd, *J* = 6.8 and 1.5 Hz, 1H), 8.49 (dd, *J* = 3.7 and 1.5 Hz, 1H), 8.13 (d, *J* = 8.0 Hz, 1H), 7.81 (s, 1H), 7.71 (d, *J* = 7.6 Hz, 1H), 7.41–7.34 (m, 2H), 7.24 (t, *J* = 7.9 Hz, 1H), 7.10–7.06 (m, 2H), 6.74–6.70 (m, *J* = 7.5 and 5.1 Hz, 2H), 4.80 (d, *J* = 5.1 Hz, 2H); ^13^C NMR (100 MHz, DMSO-d_6_) δ 149.87, 148.62, 143.72, 134.48, 134.39, 134.08, 128.54, 127.12, 126.20, 124.72, 123.64, 122.01, 120.98, 116.61, 108.85, 104.33, 37.33; LC/MS (m/z): C_17_H_14_N_4_ requires 274.32 found [M + H]^+^: 275.0.

##### *N*-(4-Methoxyphenyl)-1-(imidazo[1,2-*a*]pyrimidin-2-yl)methanamine (4c)

Flash silica gel chromatography was used to purify the crude product with the EtOAc:MeOH (10:1) solvent system. Yellow solid; 50%; mp 175–176 °C; IR (ATR) ϑ 3411, 3114, 3067, 3006, 2965, 2930, 2832, 1618, 1508, 1497, 1428, 1363, 1298, 1247, 1230, 1181, 1143, 1034, 963, 871, 807, 767, 743 cm^−1^; ^1^H NMR (400 MHz, DMSO-d_6_) δ 8.86 (dd, *J* = 6.8 and 2.0 Hz, 1H), 8.50 (dd, *J* = 4.1 and 2.0 Hz, 1H), 7.69 (s, 1H), 7.06 (dd, *J* = 6.8 and 4.1 Hz, 1H), 6.68 (d, *J* = 9.0 Hz, 2H), 6.61 (d, *J* = 9.0 Hz, 2H), 5.70 (t, *J* = 5.8 Hz, 1H), 4.52 (d, *J* = 5.8 Hz, 2H), 3.58 (s, 3H); ^13^C NMR (100 MHz, DMSO-d_6_) δ 151.66, 149.87, 148.53, 142.84, 134.05, 133.96, 121.64, 115.05, 114.23, 108.76, 55.79, 37.94; LC/MS (m/z): C_14_H_14_N_4_O requires 254.29 found [M + H]^+^: 255.0.

##### *N*-(4-(*N*^ı^*,N*^ı^-Diethylamino)phenyl)-1-(imidazo[1,2-*a*]pyrimidin-2-yl)methanamine (4d)

Flash silica gel chromatography was used to purify the crude product with the EtOAc:MeOH (10:1) solvent system. Yellow solid; 45%; mp 146–148 °C; IR (ATR) ϑ 3313, 3103, 3069, 3027, 2971, 2932, 2888, 2831, 1618, 1513, 1495, 1362, 1289, 1257, 1189, 1142, 1077, 1063, 974, 872, 807, 763 cm^−1^; ^1^H NMR (400 MHz, DMSO-d_6_) 8.88 (dd, *J* = 6.8 and 1.8 Hz, 1H), 8.51 (dd, *J* = 4.0 and 1.8 Hz, 1H), 7.70 (s, 1H), 7.06 (dd, *J* = 6.8 and 4.0 Hz, 1H), 6.60–6.54 (m, 4H), 5.48 (t, *J* = 5.2 Hz, 1H), 4.50 (d, *J* = 5.2 Hz, 2H), 3.09 (q, *J* = 6.9 Hz, 4H), 0.94 (t, *J* = 6.9 Hz, 6H); ^13^C NMR (125 MHz, DMSO-d_6_) δ 149.76, 148.45, 140.65, 140.57, 133.99, 133.79, 121.87, 116.68, 114.60, 108.66, 45.24, 38.11, 12.82; LC/MS (m/z): C_17_H_21_N_5_ requires 295.38 found [M + H]^+^: 296.0.

##### *N*-(4-Trifluoromethylphenyl)-1-(imidazo[1,2-*a*]pyrimidin-2-yl)methanamine (4e)

Flash silica gel chromatography was used to purify the crude product with the EtOAc:MeOH (10:1) solvent system. Cream solid; 50%; mp 217–218 °C. IR (ATR) ϑ 3272, 3172, 3088, 3073, 3007, 2981, 2918, 1618, 1539, 1515, 1501, 1437, 1328, 1275, 1234, 1149, 1095, 1058, 958, 822, 768, 748 cm^−1^; ^1^H NMR (400 MHz, DMSO-d_6_) 8.86 (dd, *J* = 6.7 and 1.5 Hz, 1H), 8.53 (dd, *J* = 3.8 and 1.5 Hz, 1H), 7.76 (s, 1H), 7.36 (d, *J* = 8.4 Hz, 2H), 7.11 (dd, *J* = 6.7 and 3.8 Hz, 1H), 6.89 (t, *J* = 5.2 Hz, 1H), 6.77 (d, *J* = 8.4 Hz, 2H), 4.66 (d, *J* = 5.2 Hz, 2H); ^13^C NMR (125 MHz, DMSO-d_6_) δ 150.50, 148.99, 147.51, 133.05, 132.84, 125.59 (q, *J_C-F_* = 3.6 Hz, 125.63, 125.60, 125.57, 125.54), 124.65 (q, *J_C-F_* = 270.0 Hz, 127.87, 125.72, 123.58, 121.44), 119.45, 115.34 (d, *J_C-F_* = 31.8 Hz, 115.46, 115.21), 111.24, 107.78, 35.41; LC/MS (m/z): C_14_H_11_F_3_N_4_ requires 292.26 found [M + H]^+^: 293.0.

### 2.2. ADMET analysis

ADMET analysis was carried out using the ADMETLAB 2.0 server, described previously [30]. The analysis included the following parameters: topological surface area (TPSA), permeability glycoprotein (P-gp), plasma protein binding (PPB), CYP2D6 and CYP3A4 substrate/inhibition, blood–brain barrier (BBB) permeability, human intestinal absorption (HIA), Caco2 cell permeability, and human ether-a-go-go-related gene (hERG) blocker.

### 2.3. Cell culture

MCF-7 (human breast cancer) and HUVEC (human umbilical vein endothelial) cell lines were cultured and maintained in high glucose Dulbecco’s modified Eagle medium supplemented with 10% FBS (Gibco Life Technologies, Grand Island, NY, USA), 100 U/mL penicillin, and 100 mg/mL streptomycin. The MDA–MB-231 (human breast cancer) cell line was cultured in Roswell Park Memorial Institute 1640 (RPMI 1640) medium supplemented with 10% FBS (Gibco Life Technologies), 100 U/mL penicillin, and 100 mg/mL streptomycin. All cells were passaged by trypsin EDTA when they reached 60%–70% confluence. The cells were cultivated at 37 °C in humidified air containing 5% CO_2_. All compounds were dissolved in dimethyl sulfoxide (DMSO) for the treatment processes, which did not exceed 1% in the wells.

### 2.4. Sulforhodamine B (SRB) assay

The antiproliferative effects of compounds on breast cancer cells were determined by the sulforhodamine B (SRB) assay [31]. In short, cells were seeded in a 96-well cell culture plate at a density of 5 × 10^4^ cells/mL and the plate was incubated for 24 h in a CO_2_ incubator at 37 °C. MCF-7, MDA–MB-231, and HUVEC cells were cultured for 48 h with escalating doses (1, 5, 10, 25, 50, and 100 μM) of the derivatives for determination of the IC_50_ values. After incubation, the medium was removed from the wells and each well was incubated with a 10% trichloroacetic acid (TCA) solution for fixation at +4 °C for 1 h. The wells were washed five times with distilled water (dH_2_O) to get rid of the TCA and the plate was left to dry. Fresh 0.04 (m/V) SRB dye solution was added to each well, followed by incubation for 30 min at room temperature. The plate was then dried using an air dryer after being washed four times with 1% acetic acid solution. The wells were incubated in 10 mM of the tris-base solution (pH 10.5) for 10 min on a shaker and absorbance values were measured at 565 nm with a microplate reader (Tecan Infinite® 200 PRO, Switzerland). The IC_50_ values of the compounds were recorded using the software GraphPad Prism 5.

### 2.5. Gene expression analyses by quantitative PCR

The mRNA levels of the Bax and Bcl-2 genes were analyzed using a qPCR protocol, following the procedure described previously [32]. Briefly, total RNA was extracted from MDA–MB-231 cells using the Total® RNA Purification Plus Kit, following the manufacturer’s instructions (Norgen Biotek, Thorold, ON, Canada). Subsequently, synthesis of cDNA was carried out using the high-capacity cDNA reverse transcription kit (Applied Biosystems, Foster City, CA, USA). The diluted cDNA samples were combined with specific Taqman probes and Taqman PCR Master Mix, and the amplification was recorded using Applied Biosystems 7500 Real-Time PCR systems (Applied Biosystems, Thermo Fisher Scientific) for qPCR. Changes in mRNA expression levels were determined using the comparative ΔΔCT method, with β-actin serving as an endogenous control for the qPCR analysis.

## 3. Results and discussion

### 3.1. Synthesis and characterization

**Figure 2:**
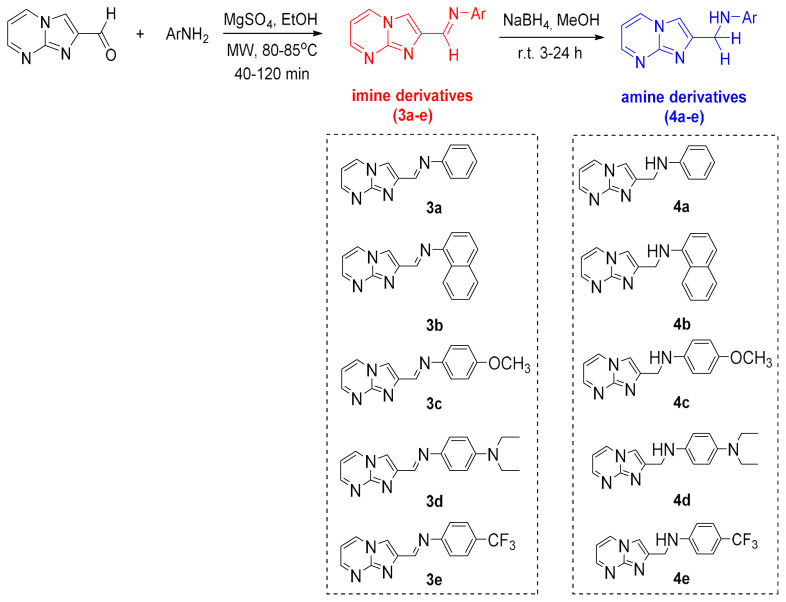
Synthesis of the target molecules, **3a**–**e** and **4a**–**e**.

One of the starting materials, imidazo[1,2-*a*]pyrimidine-2-carbaldehyde, was synthesized according to the reported data [29]. In the first step of the synthesis, imidazo[1,2-*a*]pyrimidine-2-carbaldehyde and various aromatic amines were treated in ethyl alcohol using magnesium sulfate to obtain imine derivatives (**3a**–**e**) (Figure 2). This step was conducted over 40–120 min using 200 W microwave irradiation with moderate to good yields (60%–85%). Product diversity was provided through the aromatic amines (aniline, 1-naphthylamine, *p*-anisidine, *N,N*-diethyl-*p*-phenylenediamine, and 4-(trifluoromethyl)aniline) used as starting materials. In the subsequent step, imine derivatives (**3a**–**e**) were converted to amine compounds (**4a**–**e**) in yields of 45%–70% by reduction reaction with NaBH_4_ reducing agent in methyl alcohol at room temperature (Figure 2). Synthetic details are given in the materials and methods part. All products were verified with melting point, FT-IR, ^1^H NMR, ^13^C NMR, and MS analysis. To the best of our knowledge based on the Reaxys survey, the compounds synthesized (except **3a**, **b** [14]) were reported for the first time in our study.

In our previous study, we compared different experimental conditions including conventional/microwave-assisted heating, solvent, temperature, and time for the imine formation step [14]. The results showed that ethyl alcohol, 80 °C, or toluene, 120 °C, had optimal solvent/temperature pairs. Moreover, microwave energy has prominent advantages such as short reaction times, fewer by-products, and higher yields as heating options on imine formation. In particular, the reaction time was notably decreased from 6–36 h to 40–120 min by applying microwave irradiation.

According to the FT-IR spectra of compounds **3a**–**e**, the characteristic imine group (C=N) was observed at 1626–1617 cm^−1^. After reduction of the imine group to an amine group, the secondary amine N–H vibration of compounds **4a**–**e** was determined in the range of 3411–3272 cm^−1^. Imidazo[1,2-*a*]pyrimidine heterocyclic has the characteristic one singlet and three doublet of doublets peaks in the aromatic region of the ^1^H NMR spectrum. The proton resonance signal of the imine (HC=N) group in compounds **3a**–**e** was detected as a singlet peak in the aromatic region. In the ^1^H NMR spectra of the reduction products (**4a**–**e**), while doublet hydrogen signals with 2H integral in the range of 4.80–4.50 ppm belong to the C**H****_2_**-NH, triplet hydrogen signals with 1H integral in the range of 6.89–5.48 ppm belong to the CH_2_-N**H** of amine. The ^13^C NMR chemical shift value of **C**H_2_-NH of the amine derivatives (**4a**–**e**) was assigned in the range of 38.11–35.41 ppm. Further, the calculated and measured m/z values of the products (**3a**–**e** and **4a**–**e**) were confirmed by LC-MS data. The ^1^H NMR, ^13^C NMR, and MS spectra of the compounds synthesized (**3c**–**e** and **4a**–**e**) are given in the Supporting Information part.

### 3.2. In silico ADMET analysis

In silico ADMET testing is widely used in the field of drug development to predict how a drug candidate will be absorbed, distributed, metabolized, and excreted, and whether it will have any toxicity. ADMET is a crucial step in evaluating the pharmacokinetics and pharmacodynamics of a drug candidate. ADMET tests were performed for all the compounds in the present study and the results are given in the Table, including the calculation of TPSA, BBB permeability, P-gp, PPB, CYP2D6 and CYP3A4 substrate/inhibition, Caco2 cell permeability, and HIA. The Lipinski test was also conducted, and all compounds were found to be suitable as drug candidates.

Caco2 cell permeability measures the ability of a drug to cross the intestinal barrier and enter the bloodstream, and an optimal value of Caco2 should be higher than 5.0 cm/s. The results indicated that all compounds had Caco2 values higher than 5.0 cm/s. HIA measures the percentage of an orally administered drug that is absorbed from the gastrointestinal system into the bloodstream of the human body, and the score should be less than 1.0. All the derivatives had HIA scores less than 1.0. P-gp is an efflux pump that plays a vital role in determining the ADMET properties of drugs and drug candidates [33]. The compounds showed P-gp values less than 1.0, which is the optimum value. PPB is another parameter that measures the extent to which a drug binds to plasma proteins in the bloodstream. The optimal percentage for PPB is less than 90%; however, all compounds tested had values slightly higher than 90%. TPSA is a parameter widely used in drug discovery that can predict the ability of a compound to cross biological membranes, such as the BBB, and estimate its volume of distribution in the body. TPSA is a useful predictor of oral bioavailability, with compounds that have a TPSA value of less than 140 Å^2^ being more likely to be orally bioavailable. The results showed that all compounds had TPSA values less than 140 Å^2^. The BBB is a protective barrier that separates the brain tissue from the bloodstream, and it plays a vital role in regulating the transport of substances into the brain. The results indicated that all compounds can pass through the BBB. Two of the most important enzymes in the cytochrome P450 family, CYP2D6 and CYP3A4, are responsible for the metabolism of many medications. CYP2D6 is predominantly expressed in the liver and is responsible for the metabolism and elimination of about 25% of clinically utilized medicines, including beta-blockers, opioid analgesics, antiarrhythmics, and antidepressants. On the other hand, about 50% of clinically used medications are metabolized by CYP3A4, which is the most prevalent cytochrome P450 enzyme in the liver [34]. According to the results, each substance in the present study has the potential to be both a substrate and an inhibitor of the two enzymes. A drug that prevents the potassium channels linked to the hERG is known as a hERG blocker. By regulating the movement of potassium ions, hERG channels are in charge of governing the electrical activity of the heart. They are essential for preserving the heart’s regular rhythm. Drugs can lengthen the cardiac action potential by inhibiting hERG channels, which delay the repolarization of the heart muscle. The hERG blockage score should be less than 1.0. According to the results, all compounds have a hERG blockage score of less than 1.0.

In general, the above-mentioned analyses were conducted to evaluate the efficacy and safety of the compounds as potential drug candidates. The results of the analysis could provide valuable insights into the pharmacokinetic and pharmacodynamic properties of the compounds, including their ability to interact with biological targets and their potential toxicity. Based on the ADMET analysis, every imidazo[1,2-*a*]pyrimidine derivative possesses drug-like properties except for the PPB parameter, which slightly exceeded 90%. To further investigate the potential of these compounds, in silico analyses were followed by in vitro cytotoxicity experiments. By combining in silico and in vitro analyses, it is possible to obtain a comprehensive understanding of the potential of the imidazo[1,2-*a*]pyrimidine derivatives as drug candidates, which can result in compounds being developed further.

### 3.3. Cytotoxic activity of compounds 3a–e and 4a–e

**Figure 3:**
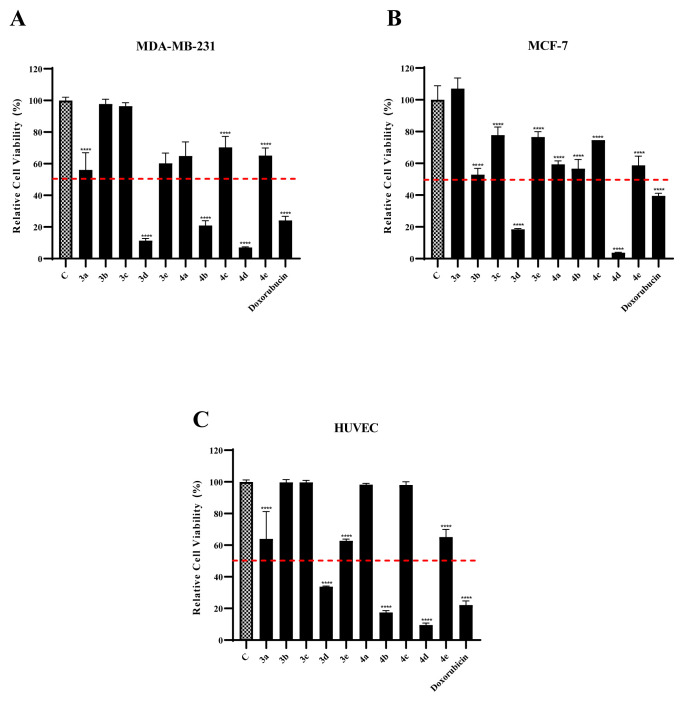
Screening of the cytotoxic effects of imidazo[1,2-*a*]pyrimidine derivatives on **(A)** MDA–MB-231, **(B)** MCF-7, and **(C)** HUVEC cells. ***p < 0.0001.

The in vitro antiproliferative activity of the synthesized compounds against the cancer cell lines was investigated since the imidazo[1,2-*a*]pyrimidine skeleton-containing compounds are known to show antiproliferative activities against cancer cells, including breast cancer [1,4,8]. It is also known that breast cancer is one of the most malignant tumor kinds and consists of 25% of cancer occurrences seen in women [24,25]. To explore the antiproliferative activity of imidazo[1,2-*a*]pyrimidine derivatives on breast cancer distinctly, we used the MCF-7 cell line, which has a functional estrogen receptor and is noninvasive; and the triple negative MDA–MB-231 cell line, which is hormone independent and more aggressive. The HUVEC cell line was used as healthy control cells to illustrate the selectivity of the derivatives for malignant tissues. The cytotoxic effects of the compounds on cell viability were assessed by SRB assay. According to the results, of all the compounds, **3d** and **4d** showed the greatest inhibition on cell proliferation of the MCF-7 and MDA–MB-231 cell lines at 100 μM dose as shown in Figure 3.

**Figure 4:**
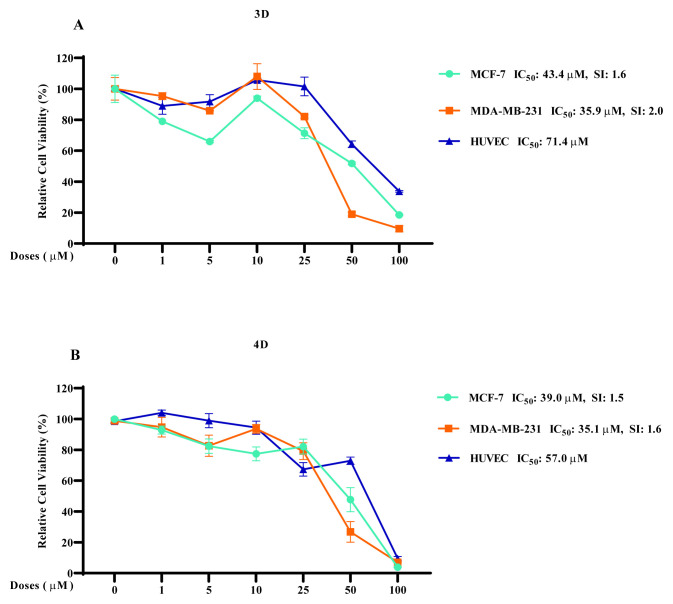
IC_50_ values and SI of **(A)** compound **3d** and **(B)** compound **4d** on MCF-7, MDA–MB-231, and HUVEC cells.

Breast cancer cells and HUVEC cells were treated with increasing doses of compounds **3d** and **4d** for IC_50_ value evaluation. As shown in Figure 4, compounds **3d** and **4d** showed inhibition on MCF-7 cells with IC_50_ values of 43.4 and 39.0 μM, and on MDA–MB-231 cells with IC_50_ values of 35.9 and 35.1 μM, respectively. The same compounds were less toxic against HUVEC endothelial cells, with an IC_50_ value for compound **3d** of 71.4 and for compound **4d** of 57.0 μM. Selectivity indexes (SIs) of the compounds were also investigated and are given in Figure 4. The SIs of compound **3d** were 1.6 for MCF-7 and 2.0 for MDA–MB-231 cells, whereas the SIs of compound **4d** were 1.5 for MCF-7 and 1.6 for MDA–MB-231 cells.

Overall, the results suggest that compounds **3d** and **4d** selectively inhibit both breast cancer cell lines in low doses, with a slightly better inhibition of the aggressive cancer cell line MDA–MB-231.

### 3.4. Effects of compounds 3d and 4d on the relative mRNA expression level of Bax/Blc-2 ratio determined by qPCR analysis

The activity of compounds **3d** and **4d** was investigated for their effectiveness against breast cancer. This part of the study focused on understanding the apoptotic mechanisms at the gene level.

**Figure 5:**
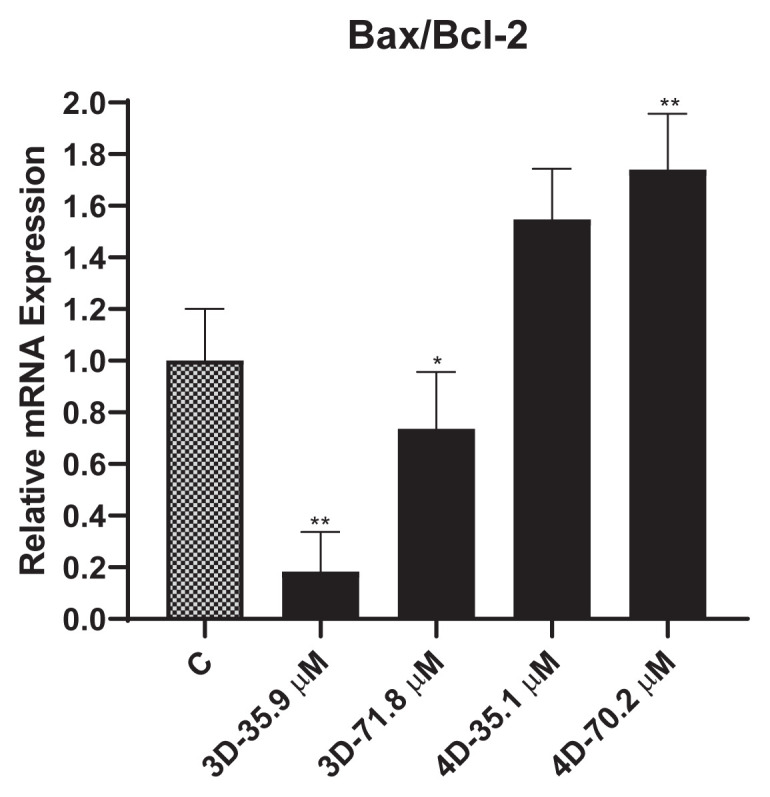
Effects of compounds **3d** and **4d** on Bax/Bcl-2 ratio at mRNA expression levels in the MDA–MB-231 cell line. *p < 0.02, **p < 0.005.

The Bax/Bcl-2 ratio is an important determinant of a cell’s susceptibility to apoptosis, with a higher ratio indicating a greater likelihood of apoptosis. For the analysis, the compounds (**3d** and **4d**) were applied to MDA–MB-231 cells at IC_50_ and IC_50_ × 2 doses. As shown in Figure 5, compound **3d** did not have significant effects on the Bax/Bcl-2 ratio at either dose. However, compound **4d** increased the Bax/Bcl-2 ratio 1.5-fold at the 35.1 μM dose and 1.8-fold at the 70.2 μM dose. Considering the overall results, the findings suggest that IC_50_ × 2 doses of compound **4d** may have moderate potential to induce apoptotic cell death in MDA–MB-231 cells.

### 3.5. Structure–activity relationship

**Figure 6:**
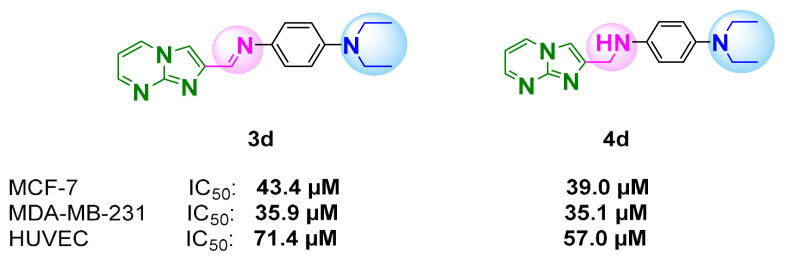
The structures of the most potent anticancer compounds, **3d** and **4d**.

It is known that imidazo[1,2-*a*]pyrimidines, which have nitrogen-containing heterocyclic skeletons, show a broad range of medicinal applications, especially anticancer activity [1,4,8]. When the structure–activity relationship (SAR) was evaluated for all the compounds, having a nitrogenous electron-donating group (–N(CH_2_CH_3_)_2_) at the 4-position of the phenyl ring in compounds **3d** and **4d** increased the cytotoxic activity on breast cancer lines (Figure 6). The oxygenated electron-donating unit (–OCH_3_, **3c**–**4c**) and fluorinated electron-withdrawing unit (–CF_3_, **3e**–**4e**) on the phenyl ring and nonsubstituted phenyl and naphthyl groups (**3a**, **b** and **4a**, **b**) did not show a remarkable inhibitory effect on the proliferation of cancer lines. With the reduction conversion of the imine group (**3d)** to an amine group (**4d**) cytotoxic efficiency was slightly increased in the MCF-7 cancer cells, while no change was observed in the MDA–MB-231 cancer cells. The possible reason for this was that with the change in structure as a result of disruption of planarity and conjugation at the molecule, the amine group (CH_2_-NH) has various interactions with different cancer-related biomarkers in the anticancer mechanism. In addition, both compounds draw attention due to their slightly better antiproliferative effect on the more aggressive cancer cell line, MDA–MB-231. It can be concluded that compound **3d** is the best cytotoxic agent among all the compounds synthesized (**3a**–**e** and **4a**–**e**) for breast cancer based on IC_50_ values and selectivity indexes compared to the healthy HUVEC cells.

## 4. Conclusion

Herein we have presented the synthesis and cytotoxic screening studies of a series of imine-/amine-containing imidazo[1,2-*a*]pyrimidine compounds (**3a**–**e** and **4a**–**e**). The synthesis study consists of two steps: imine (C=N) formation from imidazo[1,2-*a*]pyrimidine-2-carbaldehyde and aromatic amine derivatives, and the reduction reaction of imine to obtain amine derivatives. The chemical structures of the products were elucidated with FT-IR, ^1^H NMR, ^13^C NMR, and LC-MS spectrometric methods. The anticancer activities of the products were determined by SRB assay in human breast cancer (MCF-7 and MDA–MB-231) and human healthy (HUVEC) cell lines. Among the 10 compounds synthesized, **3d** and **4d** derivatives were the most effective against breast cancer cell lines. In particular, compound **3d** demonstrated significant inhibition of cell proliferation, in which IC_50_ values were 43.4, 35.9, and 71.4 μM on MCF-7, MDA–MB-231, and HUVEC cells, respectively. It can be concluded that compound **3d** selectively inhibits the proliferation of breast cancer cells 1.6-fold (MCF-7) and 2.0-fold (MDA–MB-231) relative to healthy cells. Compound **4d** induced apoptosis moderately through known markers of apoptosis, Bax and Bcl-2. In future studies, to improve the cytotoxic activity on breast cancer cells, developing new imine-/amine- and imidazo[1,2-*a*]pyrimidine-containing derivatives that can be modified by bonding different nitrogen-containing electron-donating groups on the phenyl ring or linking longer alkyl amino chains between heterocyclic and phenyl rings is planned.

## Supplementary Information

Figure S1 ^1^H NMR spectrum of compound **3c**.

Figure S2 ^13^C NMR spectrum of compound **3c**.

Figure S3 MS spectrum of compound **3c**.

Figure S4 ^1^H NMR spectrum of compound **3d**.

Figure S5 ^13^C NMR spectrum of compound **3d**.

Figure S6 MS spectrum of compound **3d**.

Figure S7 ^1^H NMR spectrum of compound **3e**.

Figure S8 ^13^C NMR spectrum of compound **3e**.

Figure S9 MS spectrum of compound **3e**.

Figure S10 ^1^H NMR spectrum of compound **4a**.

Figure S11 ^13^C NMR spectrum of compound **4a**.

Figure S12 MS spectrum of compound **4a**.

Figure S13 ^1^H NMR spectrum of compound **4b**.

Figure S14 ^13^C NMR spectrum of compound **4b**.

Figure S15 MS spectrum of compound **4b**.

Figure S16 ^1^H NMR spectrum of compound **4c**.

Figure S17 ^13^C NMR spectrum of compound **4c**.

Figure S18 MS spectrum of compound **4c**.

Figure S19 ^1^H NMR spectrum of compound **4d**.

Figure S20 ^13^C NMR spectrum of compound **4d**.

Figure S21 MS spectrum of compound **4d**.

Figure S22 ^1^H NMR spectrum of compound **4e**.

Figure S23 ^13^C NMR spectrum of compound **4e**.

Figure S24 MS spectrum of compound **4e**.

## Figures and Tables

**Table:** ADMET analysis of imidazo[1,2-*a*]pyrimidine derivatives.

COMPOUNDS	CACO-2 PERMEABILITY (CM/S)	HIA	PGP (I)	PGP (S)	PPB%	TPSA	BBB	CYP3A4 INHIBITOR	CYP3A4 SUBSTRATE	CYP2D6 INHIBITOR	CYP2D6 SUBSTRATE	hERG BLOCKER	LIPINSKI
**3a**	−4.284	0.007	0.002	0.002	91.41%	42.55	0.957	0.008	0.22	0.019	0.165	0.009	Yes
**3b**	−4.437	0.007	0.015	0.008	96.02%	42.55	0.957	0.066	0.165	0.243	0.535	0.006	Yes
**3c**	−4.421	0.005	0.003	0.001	92.00%	51.78	0.964	0.089	0.394	0.088	0.783	0.016	Yes
**3d**	−4.261	0.004	0.003	0.008	92.90%	45.79	0.776	0.066	0.237	0.07	0.195	0.041	Yes
**3e**	−4.451	0.004	0.006	0.005	94.50%	42.55	0.908	0.02	0.172	0.088	0.206	0.013	Yes
**4a**	−4.365	0.031	0.001	0.453	91.60%	42.22	0.958	0.062	0.158	0.035	0.496	0.04	Yes
**4b**	−4.495	0.042	0.013	0.771	96.18%	42.22	0.955	0.589	0.127	0.701	0.854	0.15	Yes
**4c**	−4.441	0.01	0.001	0.086	92.32%	51.45	0.964	0.36	0.181	0.167	0.885	0.074	Yes
**4d**	−4.468	0.01	0.001	0.971	92.18%	45.46	0.846	0.223	0.123	0.105	0.544	0.158	Yes
**4e**	−4.518	0.006	0.007	0.422	94.19%	42.22	0.895	0.197	0.189	0.183	0.692	0.092	Yes
