# Supplementary figures and images for: Synthesis of new imine-/amine-bearing imidazo[1,2-a]pyrimidine derivatives and screening of their cytotoxic activity
Source: Turk J Chem. 2023 Oct 11;47(5):1064–74. doi: 10.55730/1300-0527.3594 (PMC10760840; doi:10.55730/1300-0527.3594)

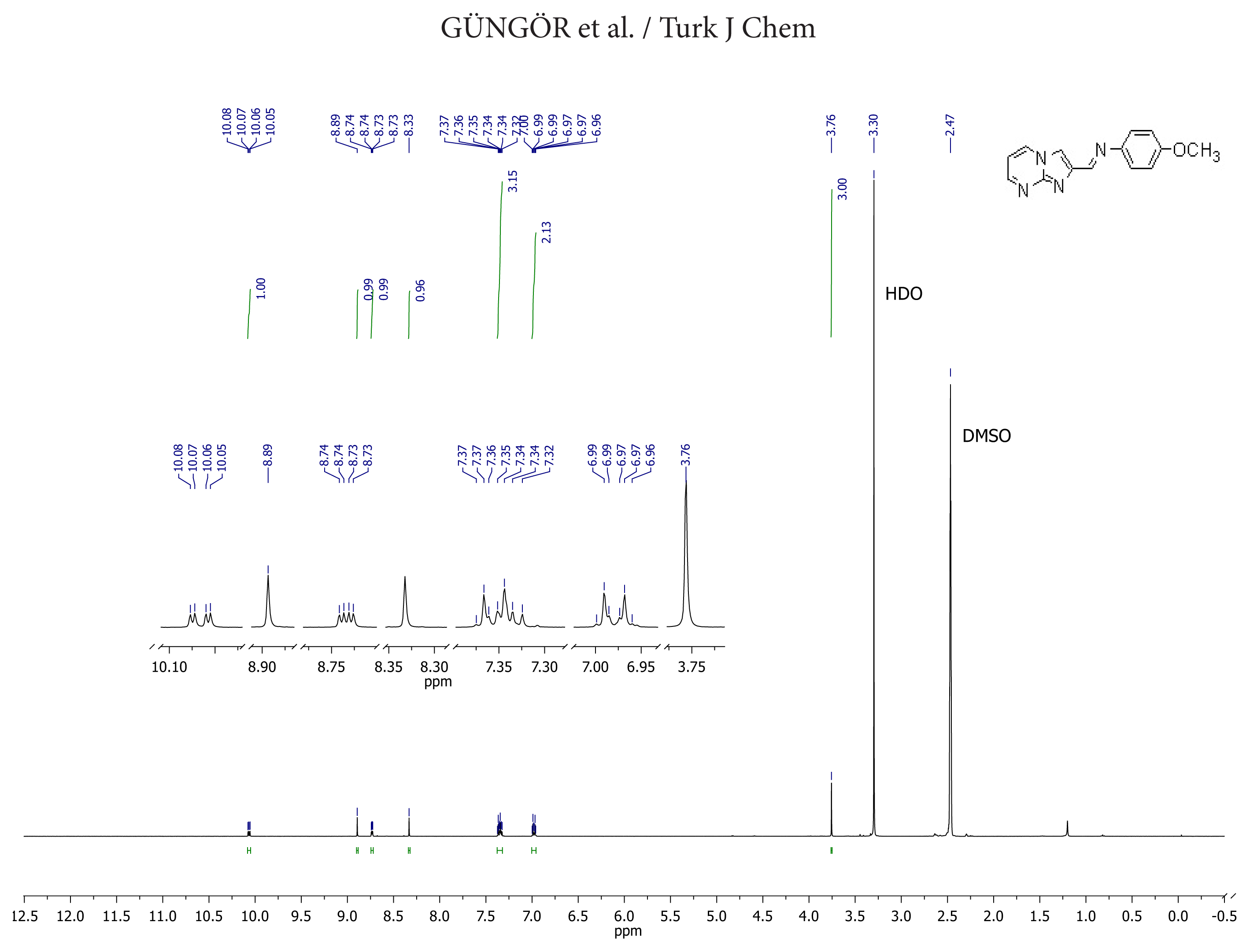

Supplement: Figure S1 — 1H NMR spectrum of compound 3c. [file turkjchem-47-5-1064s1.tif]

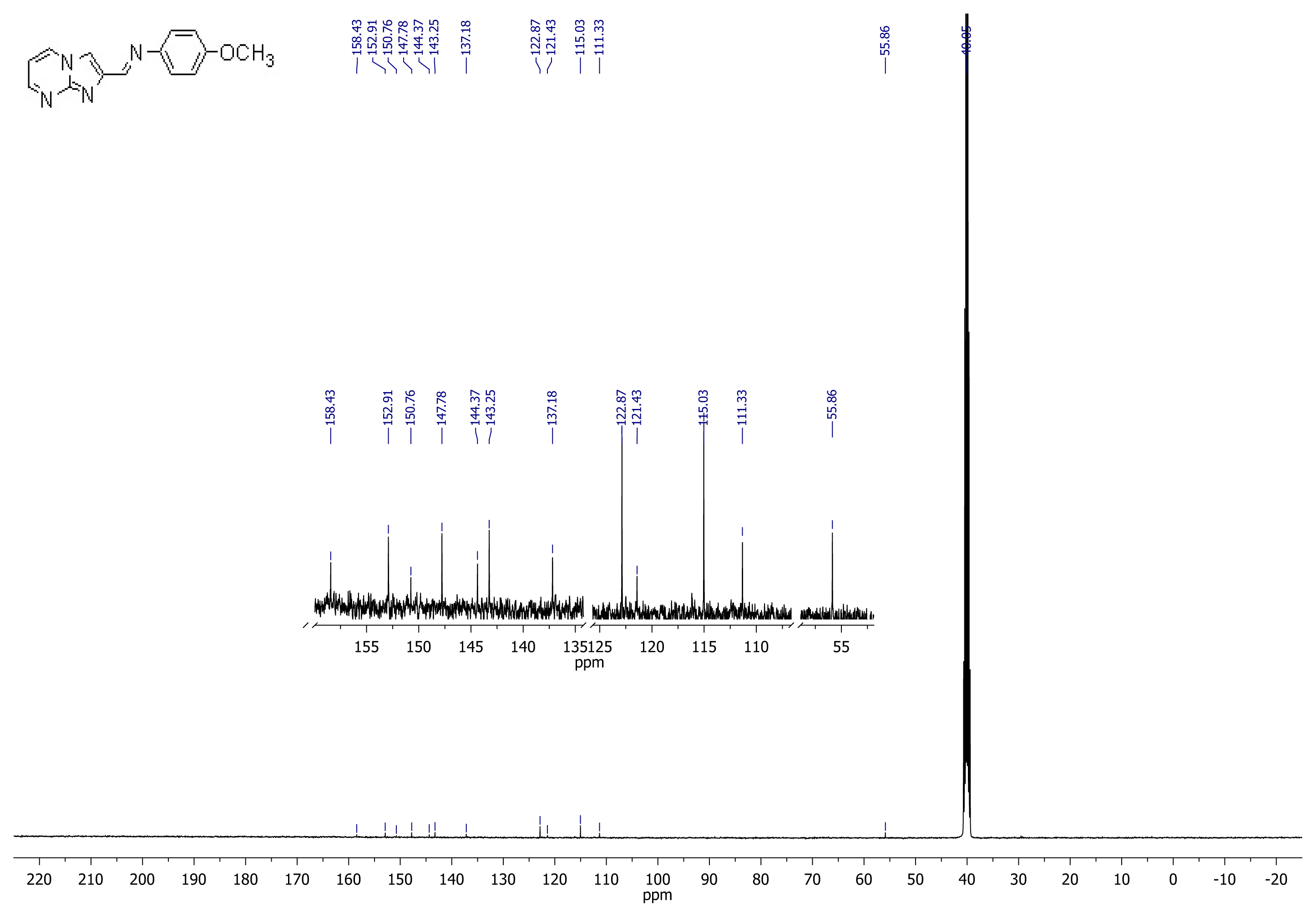

Supplement: Figure S2 — 13C NMR spectrum of compound 3c. [file turkjchem-47-5-1064s2.tif]

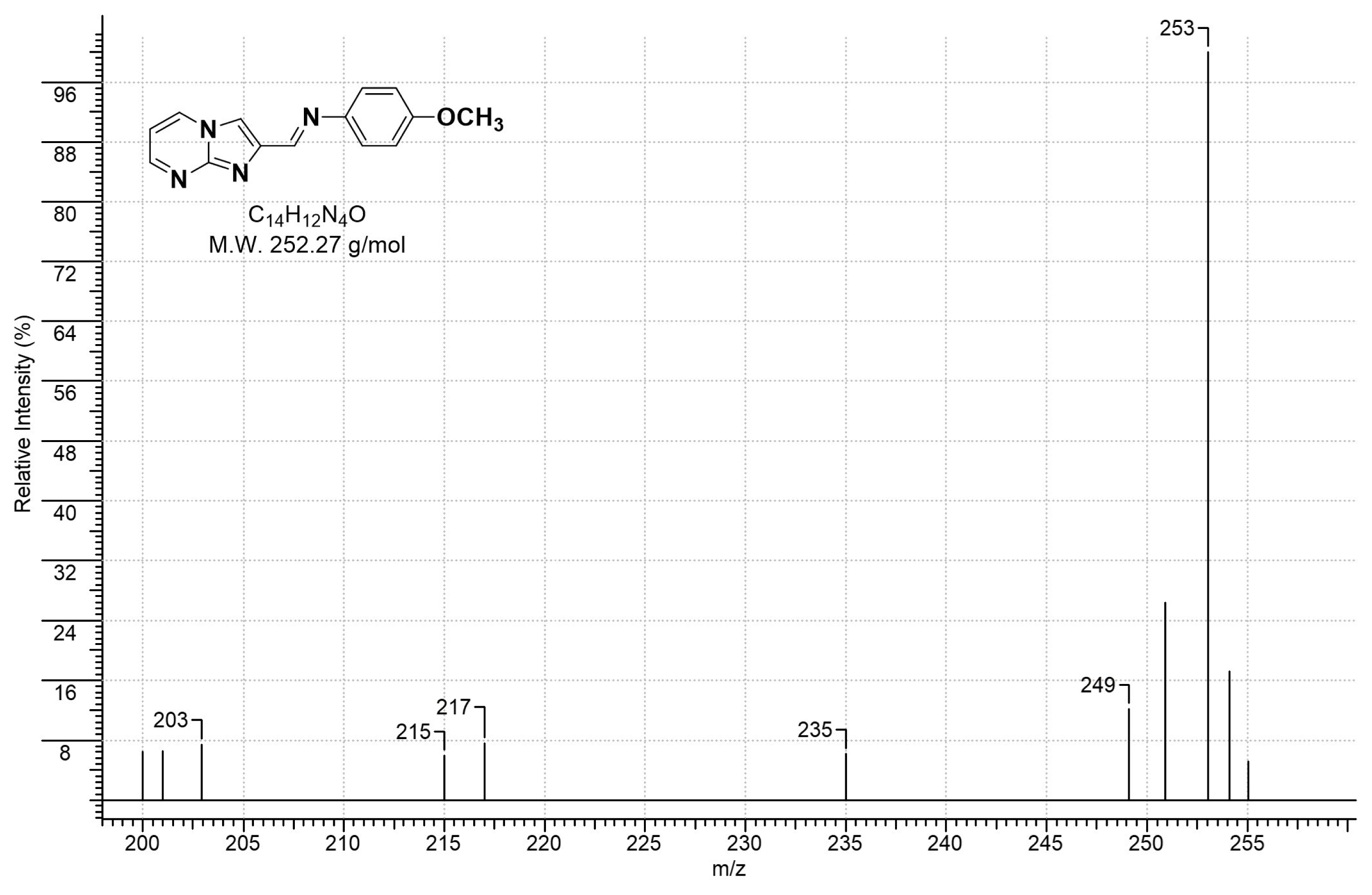

Supplement: Figure S3 — MS spectrum of compound 3c. [file turkjchem-47-5-1064s3.tif]

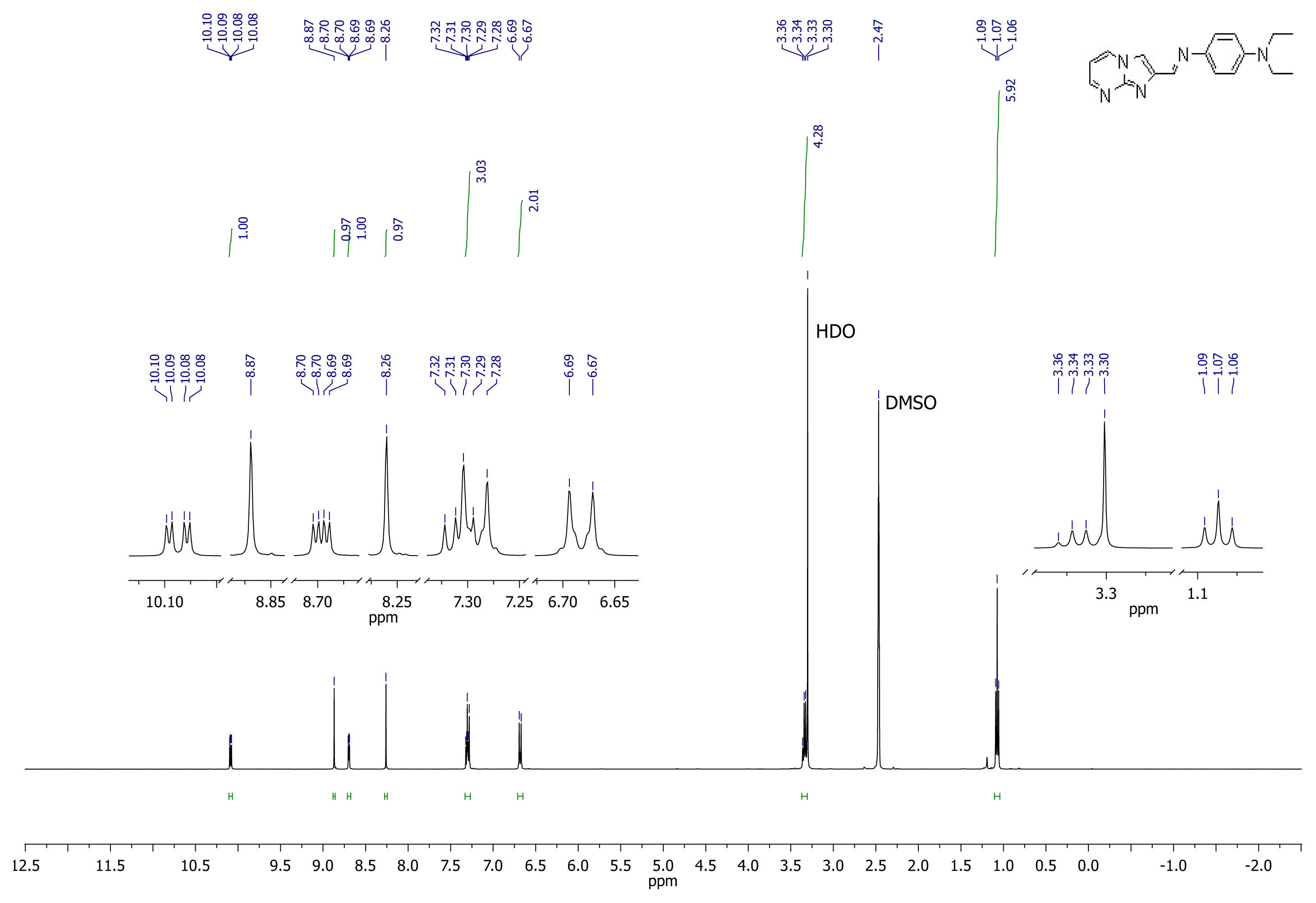

Supplement: Figure S4 — 1H NMR spectrum of compound 3d. [file turkjchem-47-5-1064s4.tif]

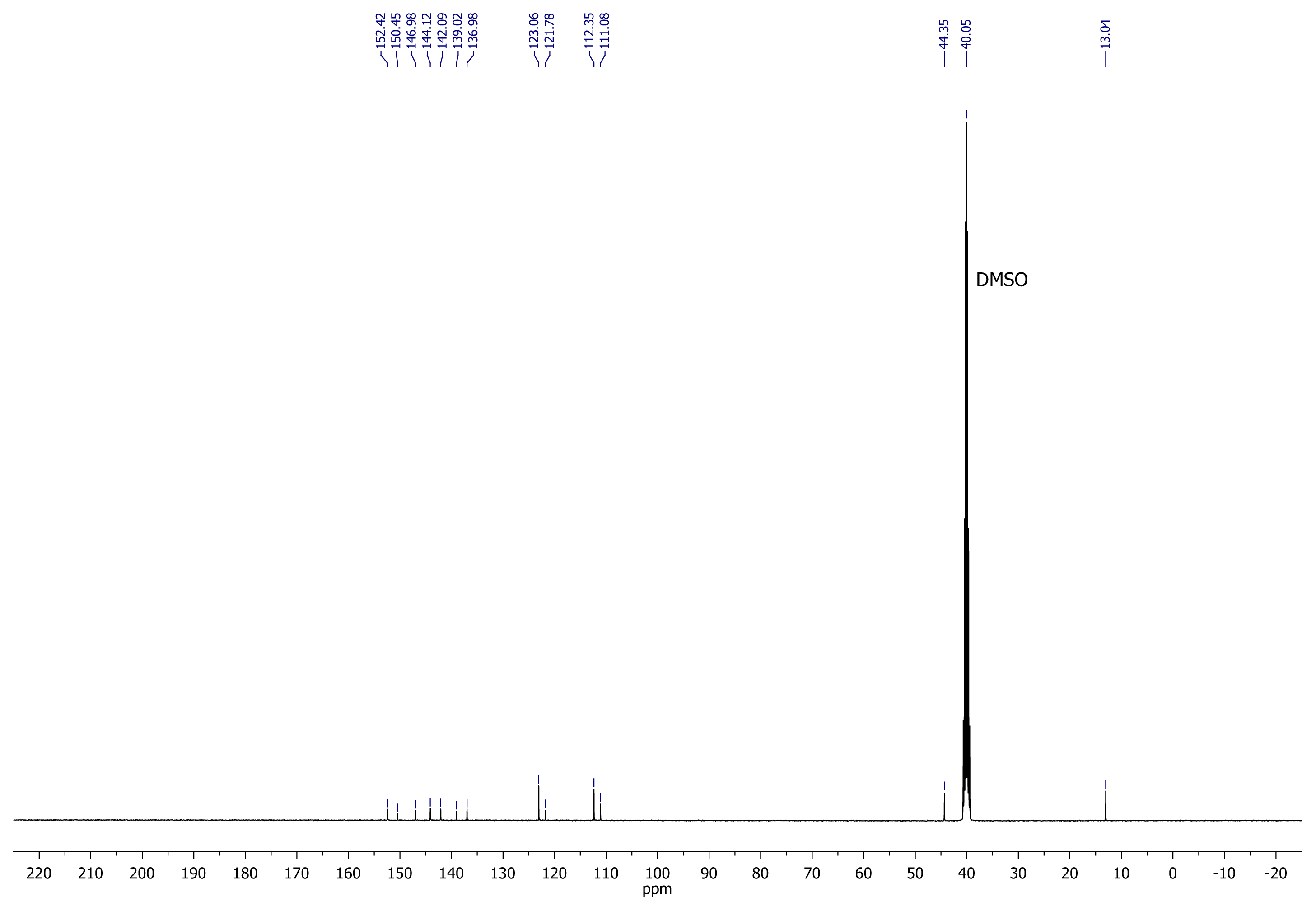

Supplement: Figure S5 — 13C NMR spectrum of compound 3d. [file turkjchem-47-5-1064s5.tif]

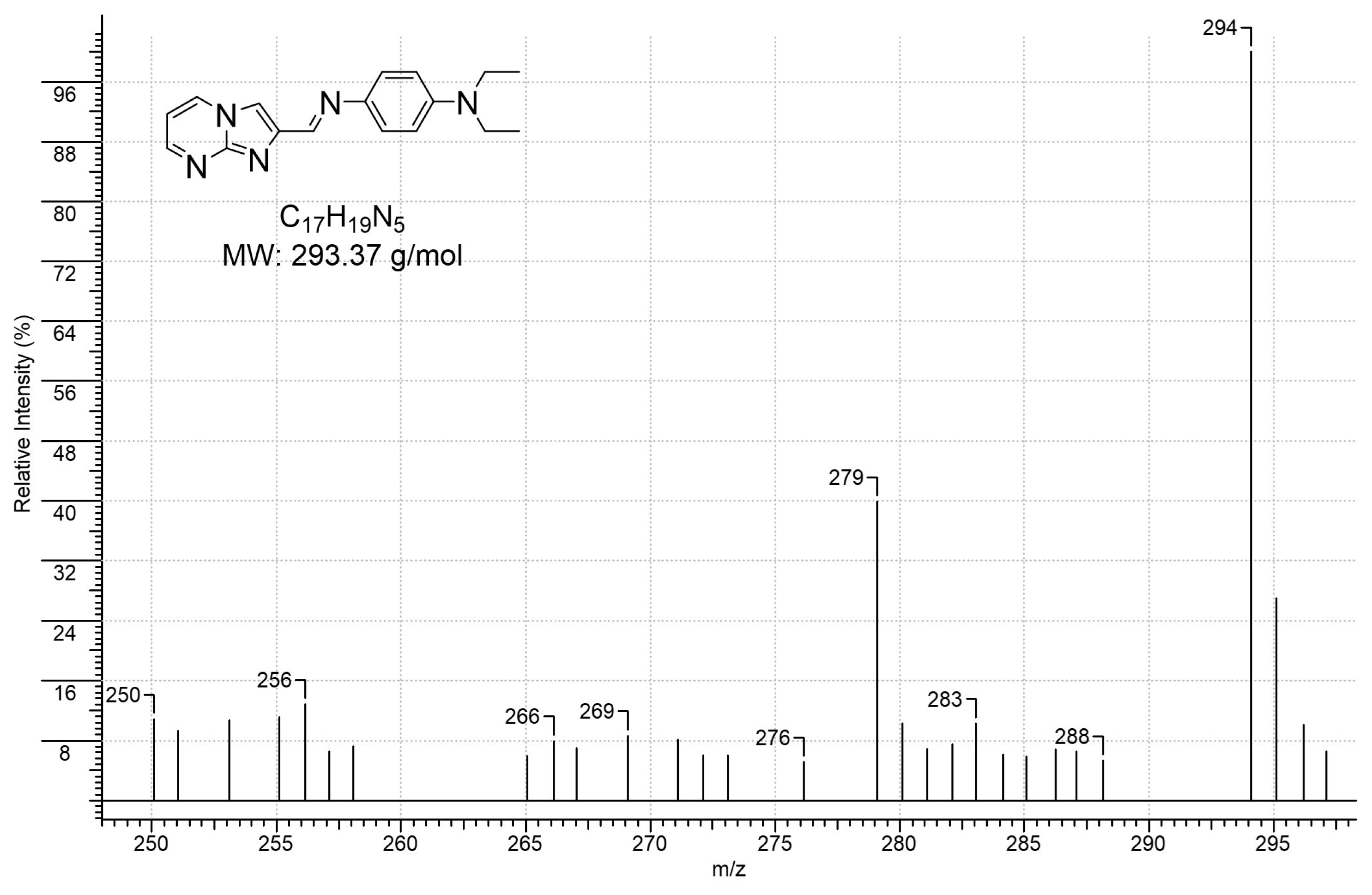

Supplement: Figure S6 — MS spectrum of compound 3d. [file turkjchem-47-5-1064s6.tif]

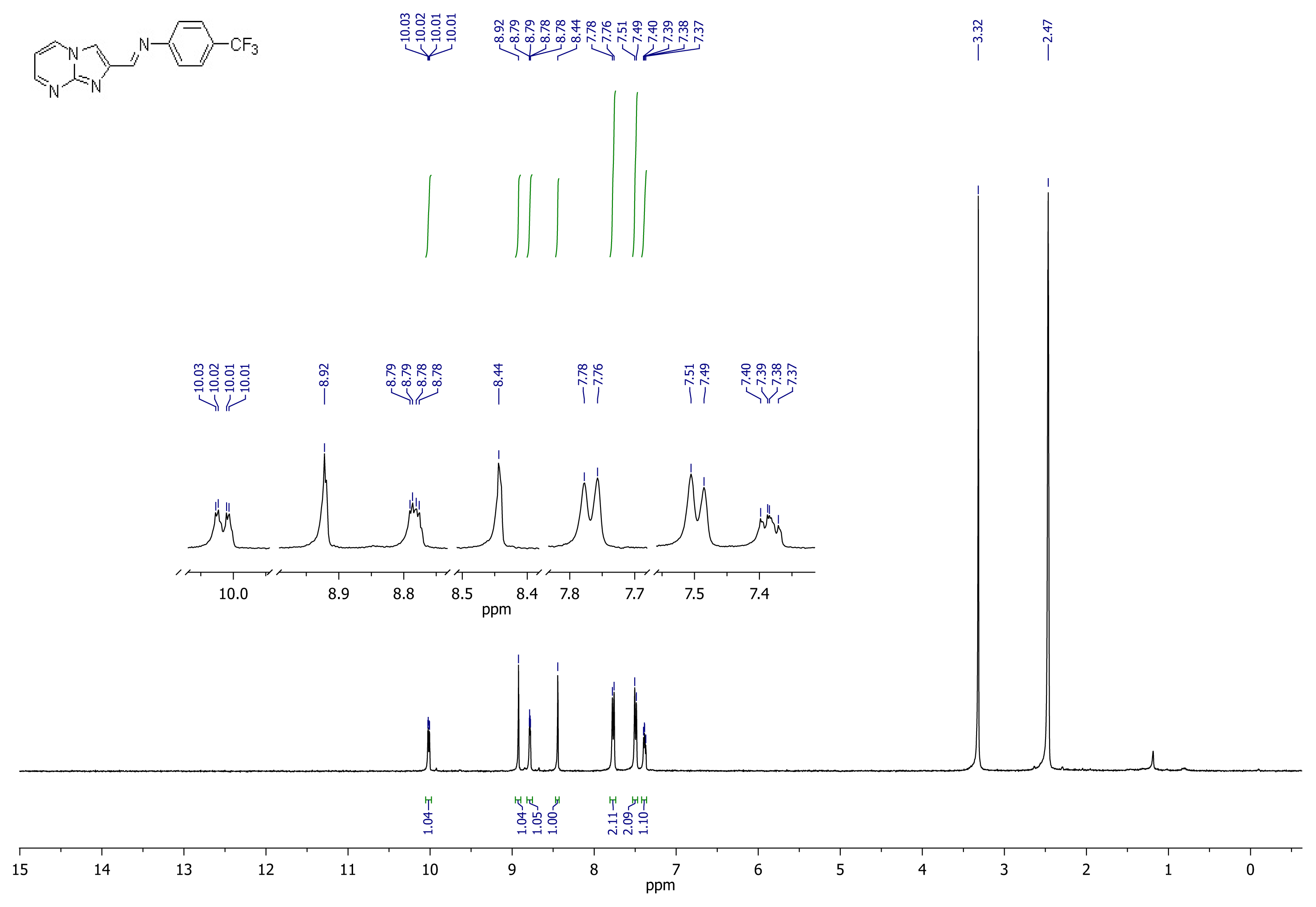

Supplement: Figure S7 — 1H NMR spectrum of compound 3e. [file turkjchem-47-5-1064s7.tif]

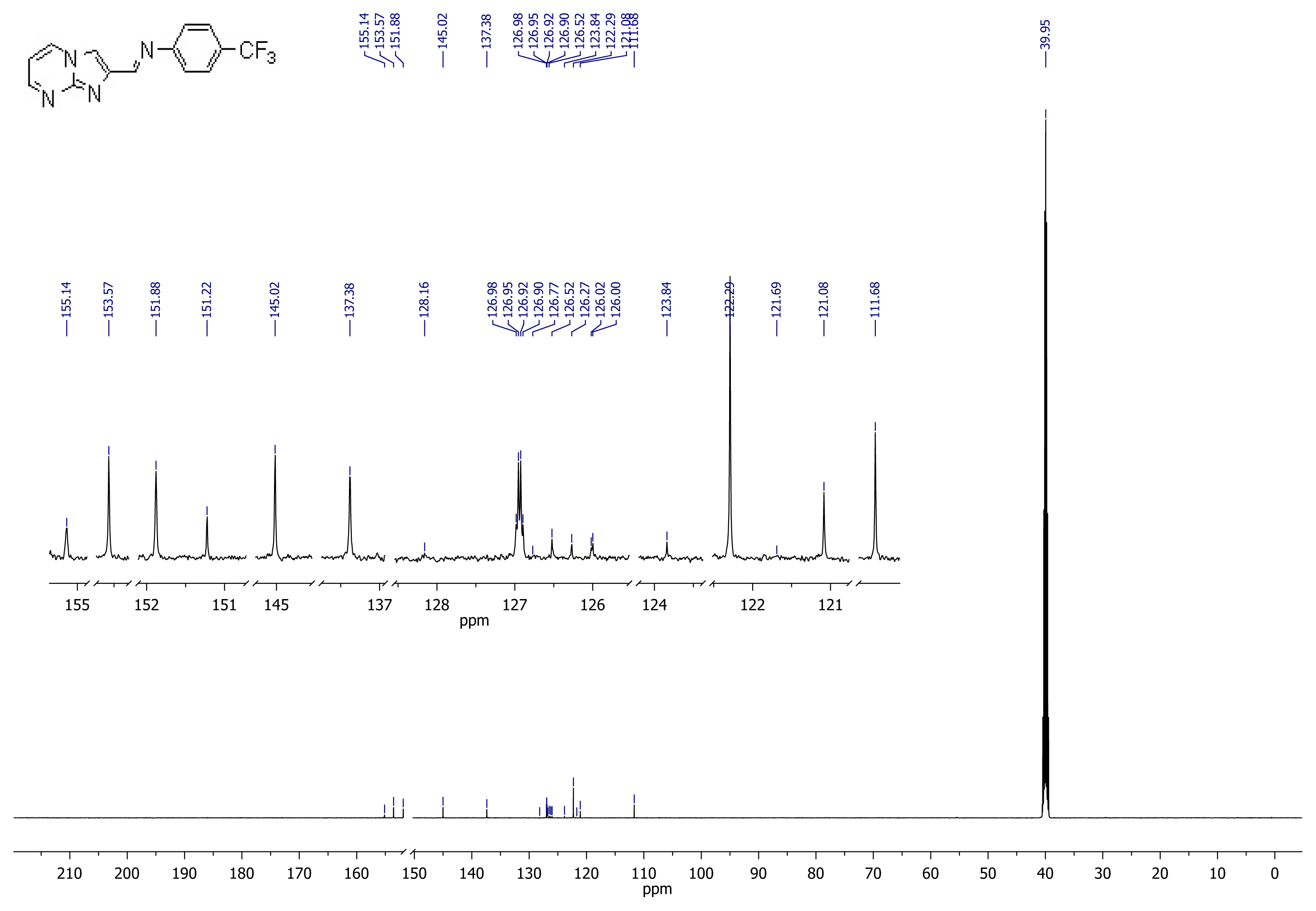

Supplement: Figure S8 — 13C NMR spectrum of compound 3e. [file turkjchem-47-5-1064s8.tif]

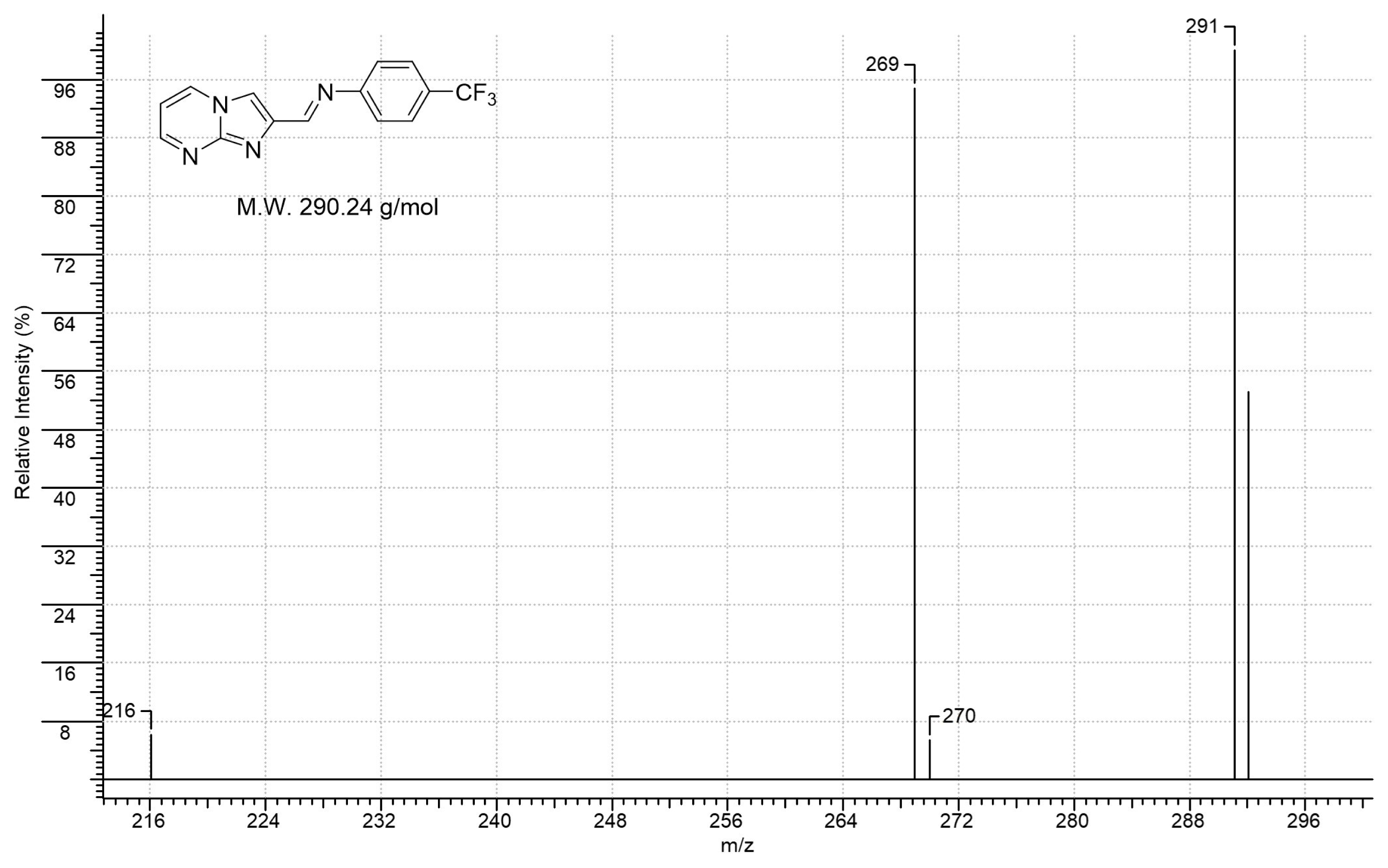

Supplement: Figure S9 — MS spectrum of compound 3e. [file turkjchem-47-5-1064s9.tif]

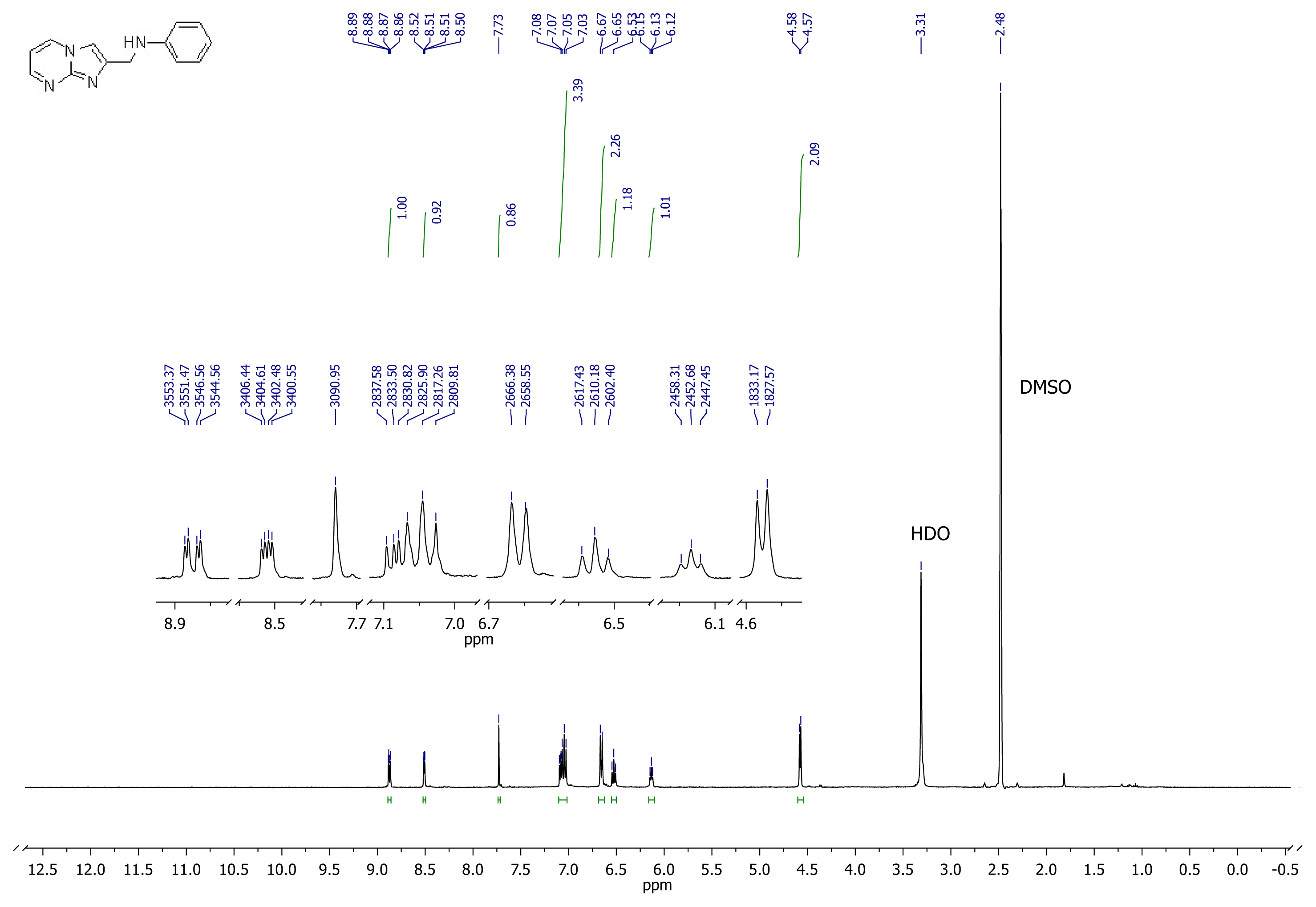

Supplement: Figure S10 — 1H NMR spectrum of compound 4a. [file turkjchem-47-5-1064s10.tif]

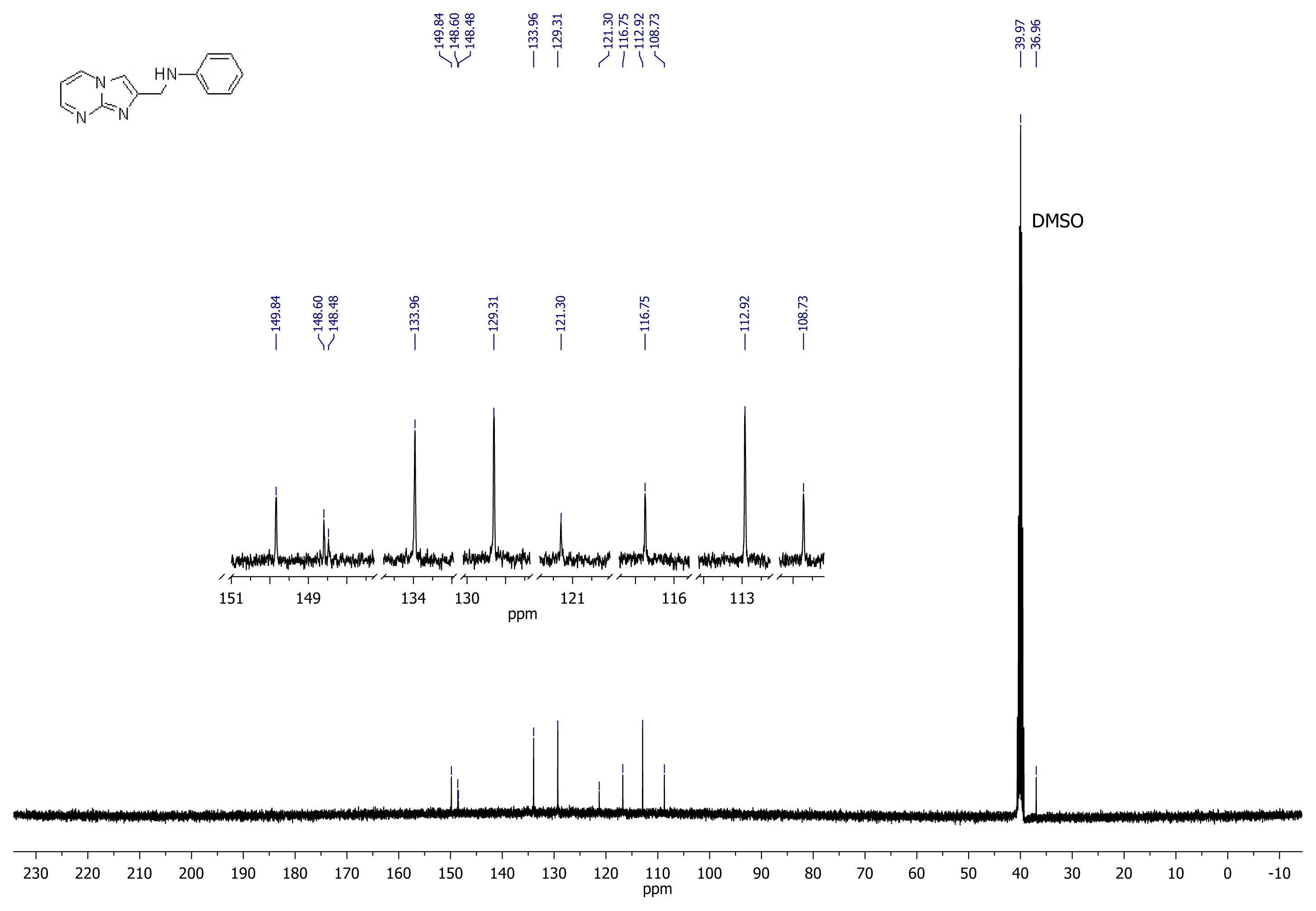

Supplement: Figure S11 — 13C NMR spectrum of compound 4a. [file turkjchem-47-5-1064s11.tif]

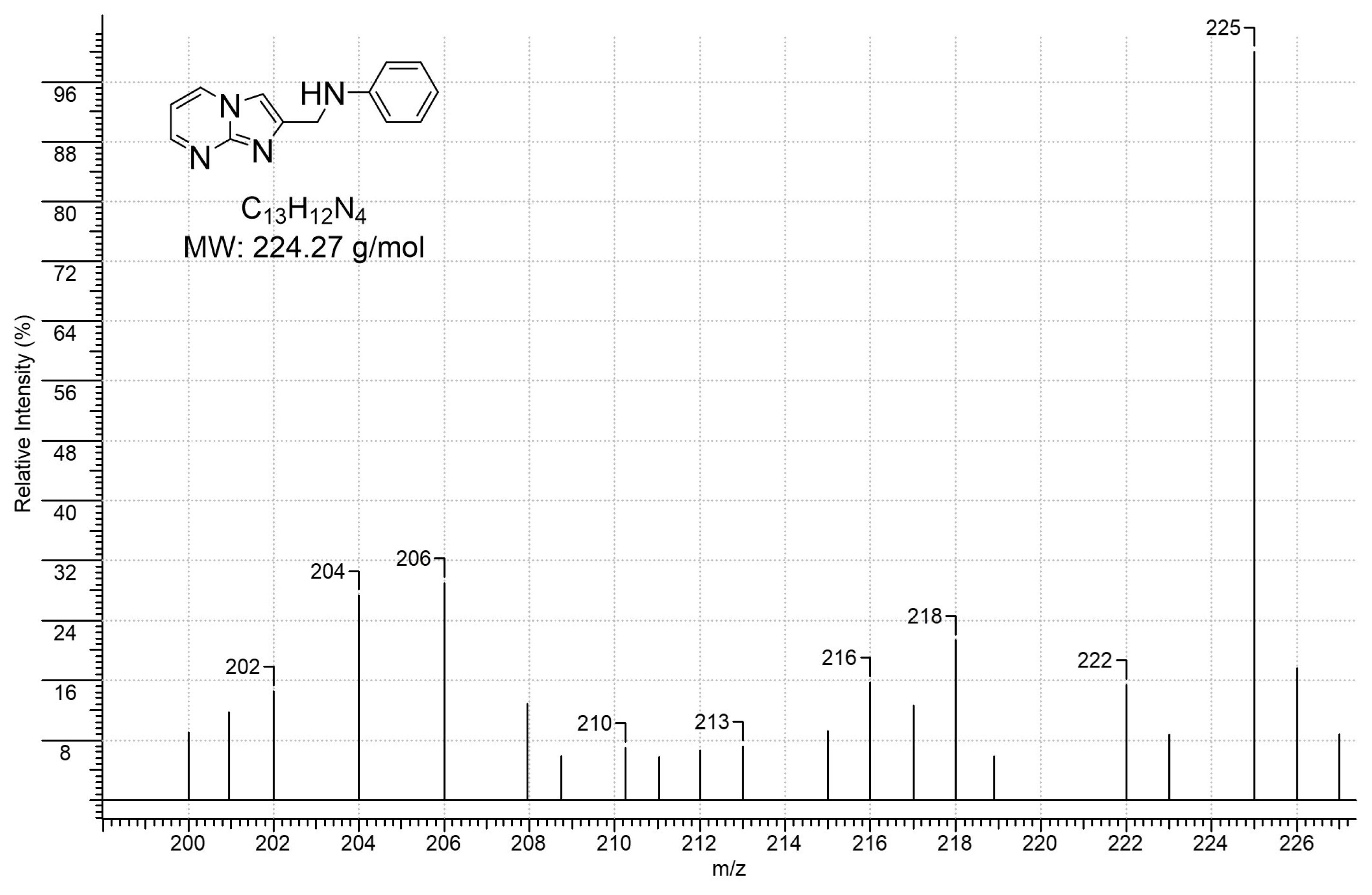

Supplement: Figure S12 — MS spectrum of compound 4a. [file turkjchem-47-5-1064s12.tif]

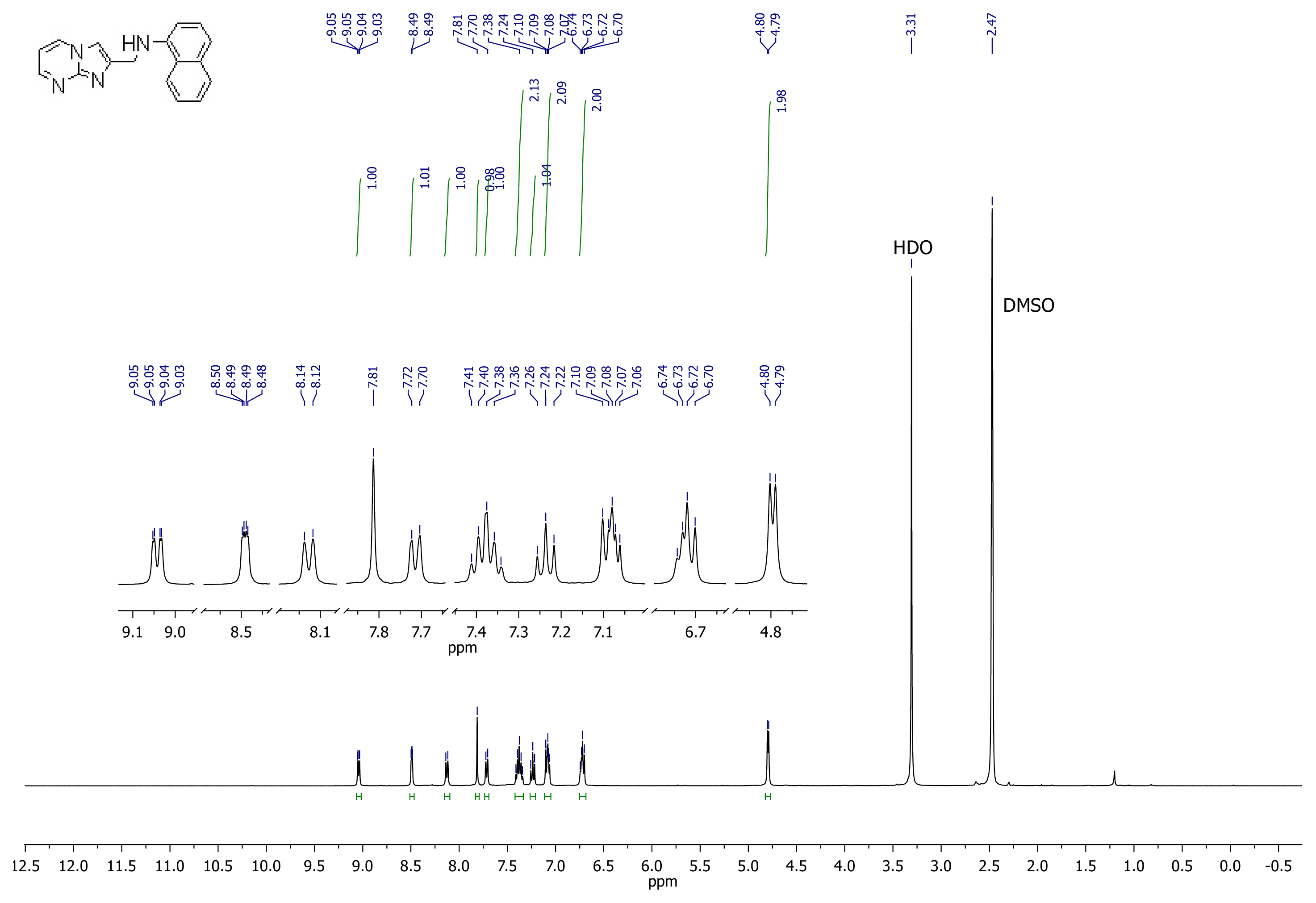

Supplement: Figure S13 — 1H NMR spectrum of compound 4b. [file turkjchem-47-5-1064s13.tif]

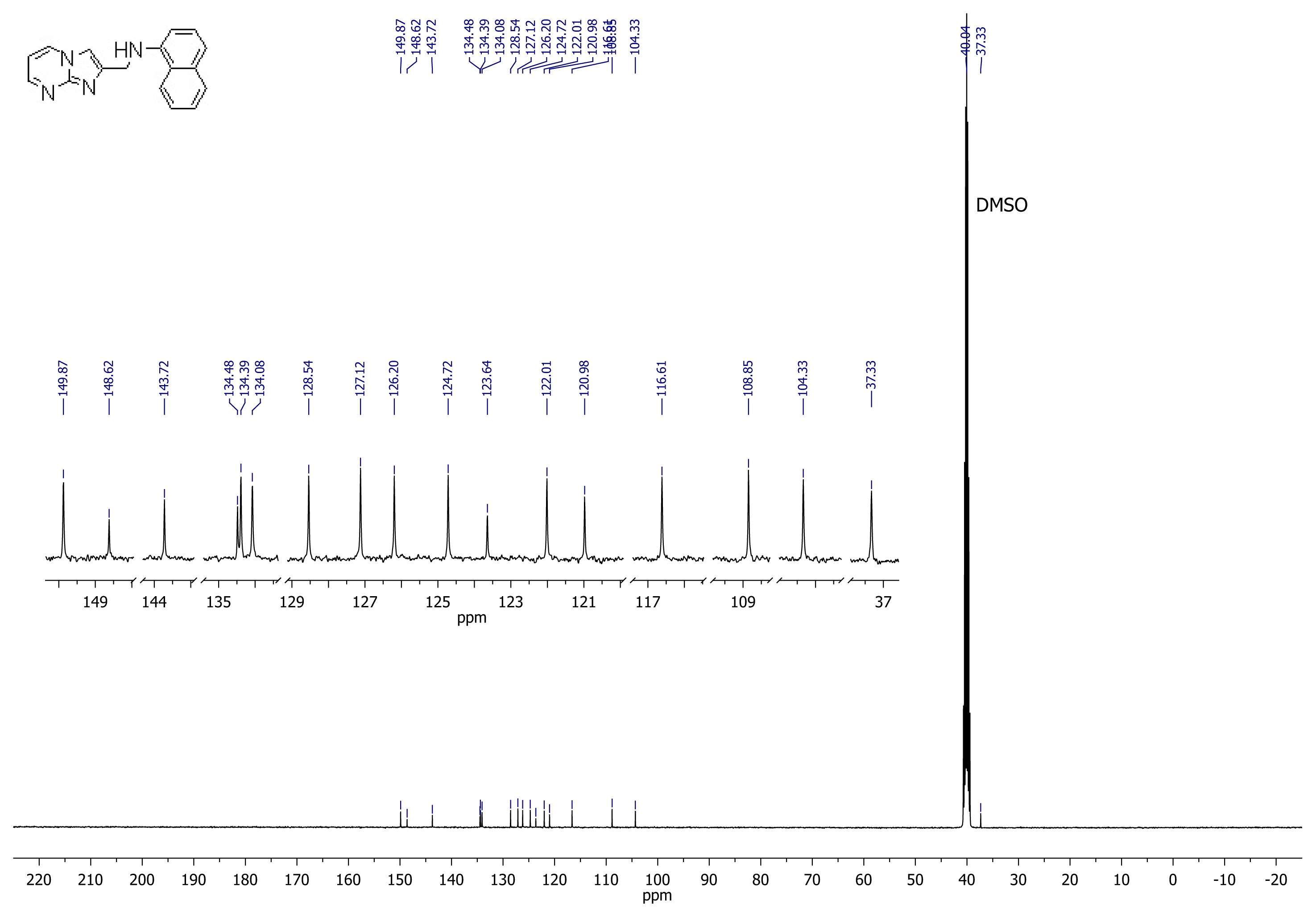

Supplement: Figure S14 — 13C NMR spectrum of compound 4b. [file turkjchem-47-5-1064s14.tif]

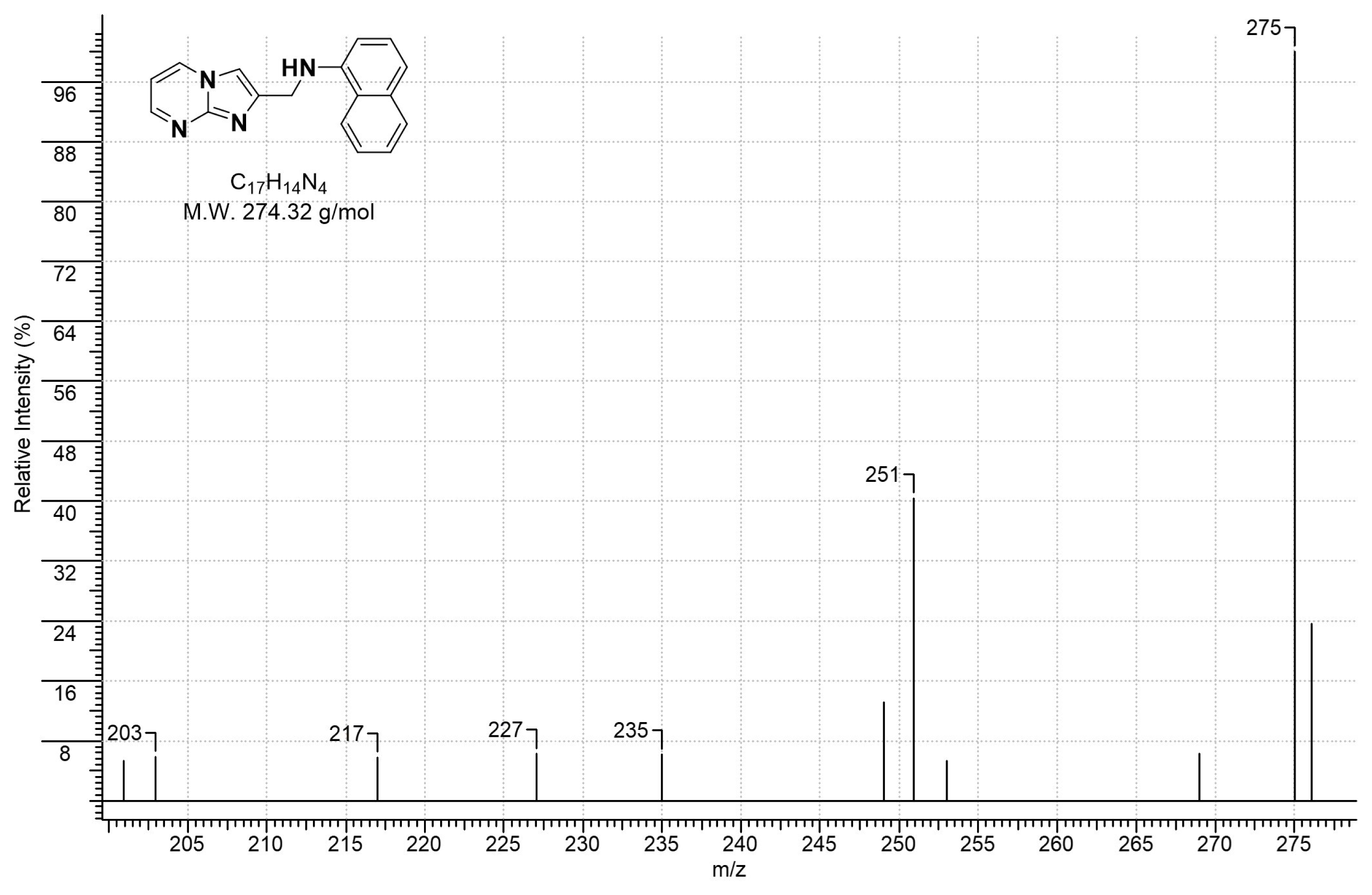

Supplement: Figure S15 — MS spectrum of compound 4b. [file turkjchem-47-5-1064s15.tif]

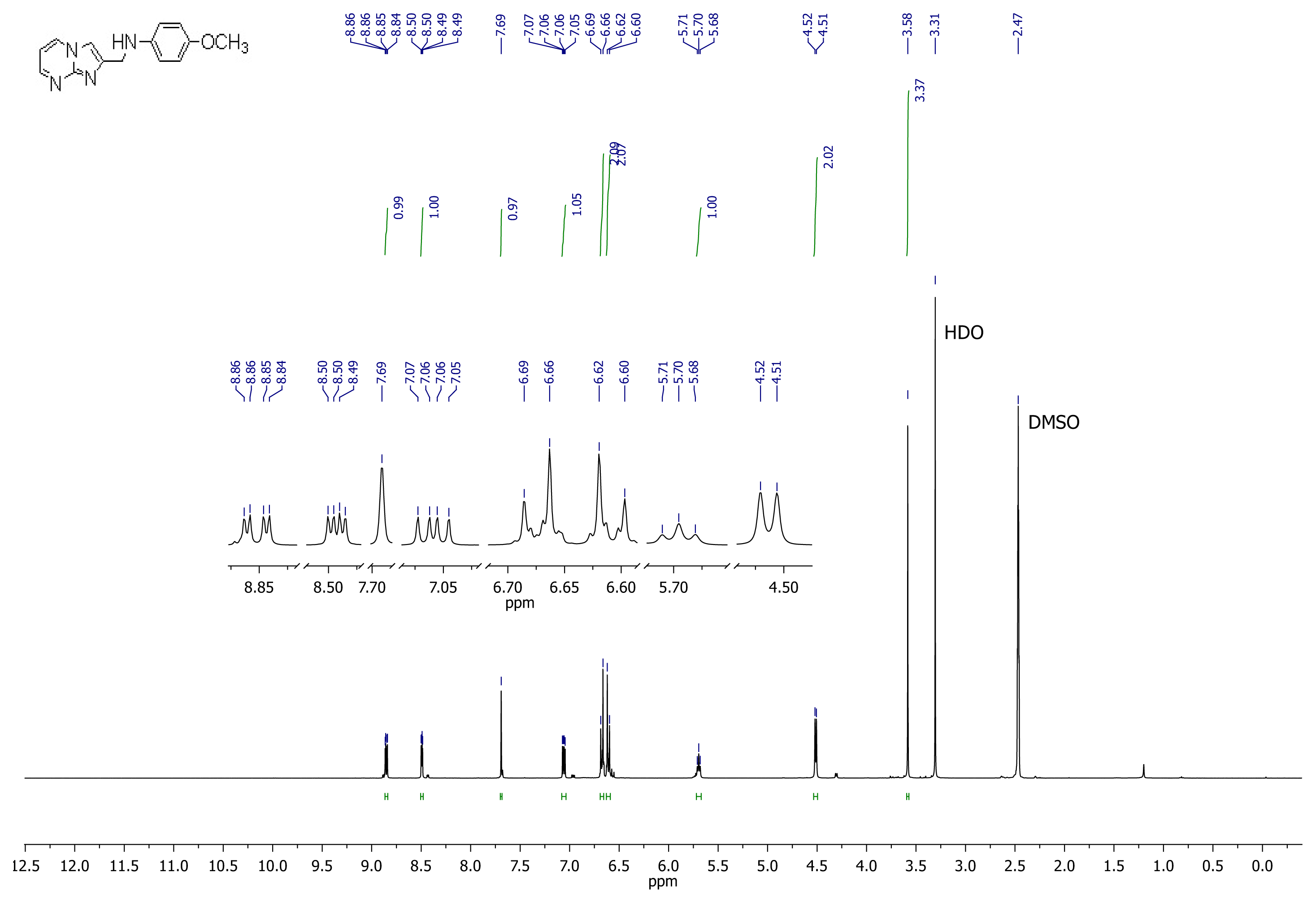

Supplement: Figure S16 — 1H NMR spectrum of compound 4c. [file turkjchem-47-5-1064s16.tif]

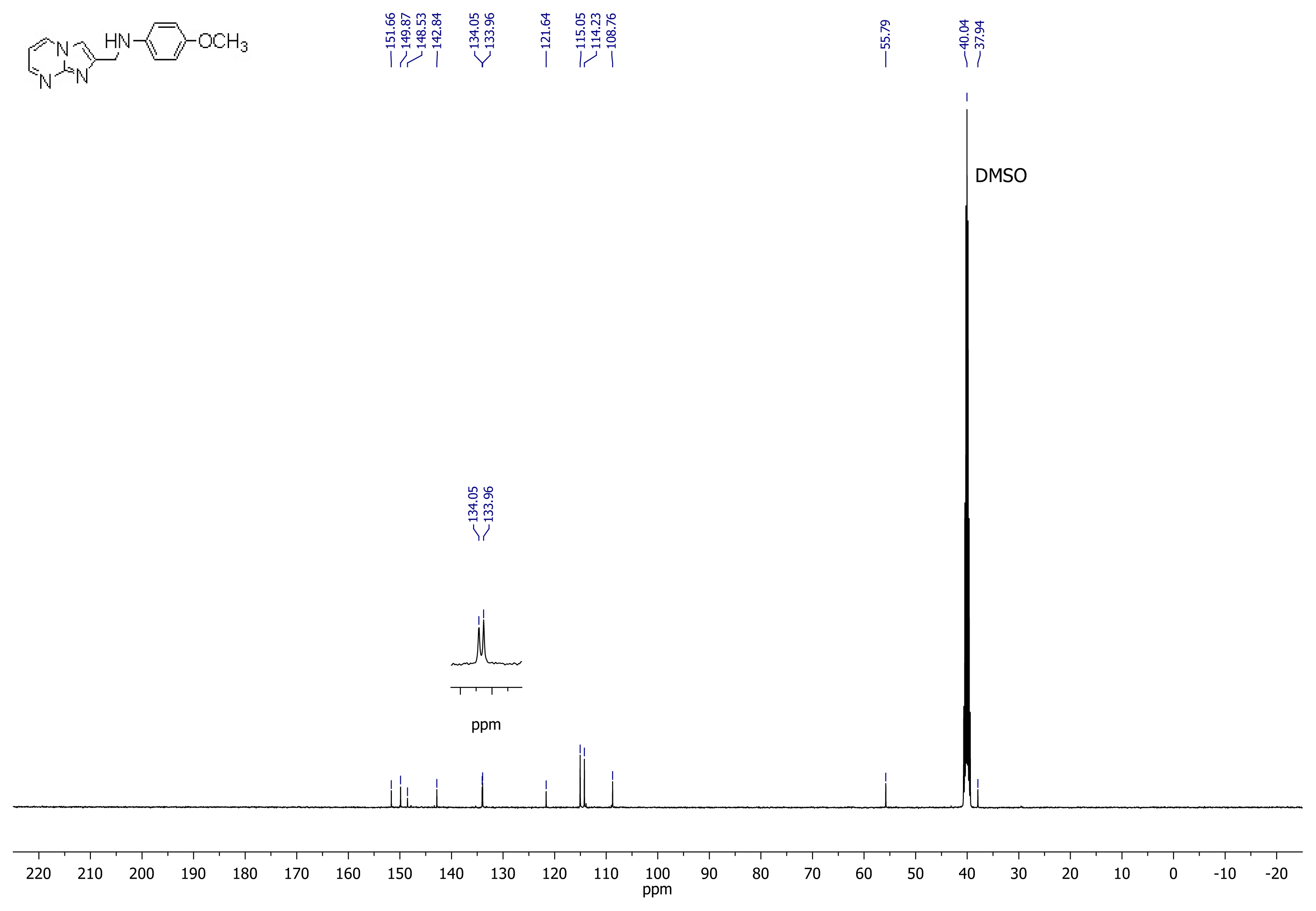

Supplement: Figure S17 — 13C NMR spectrum of compound 4c. [file turkjchem-47-5-1064s17.tif]

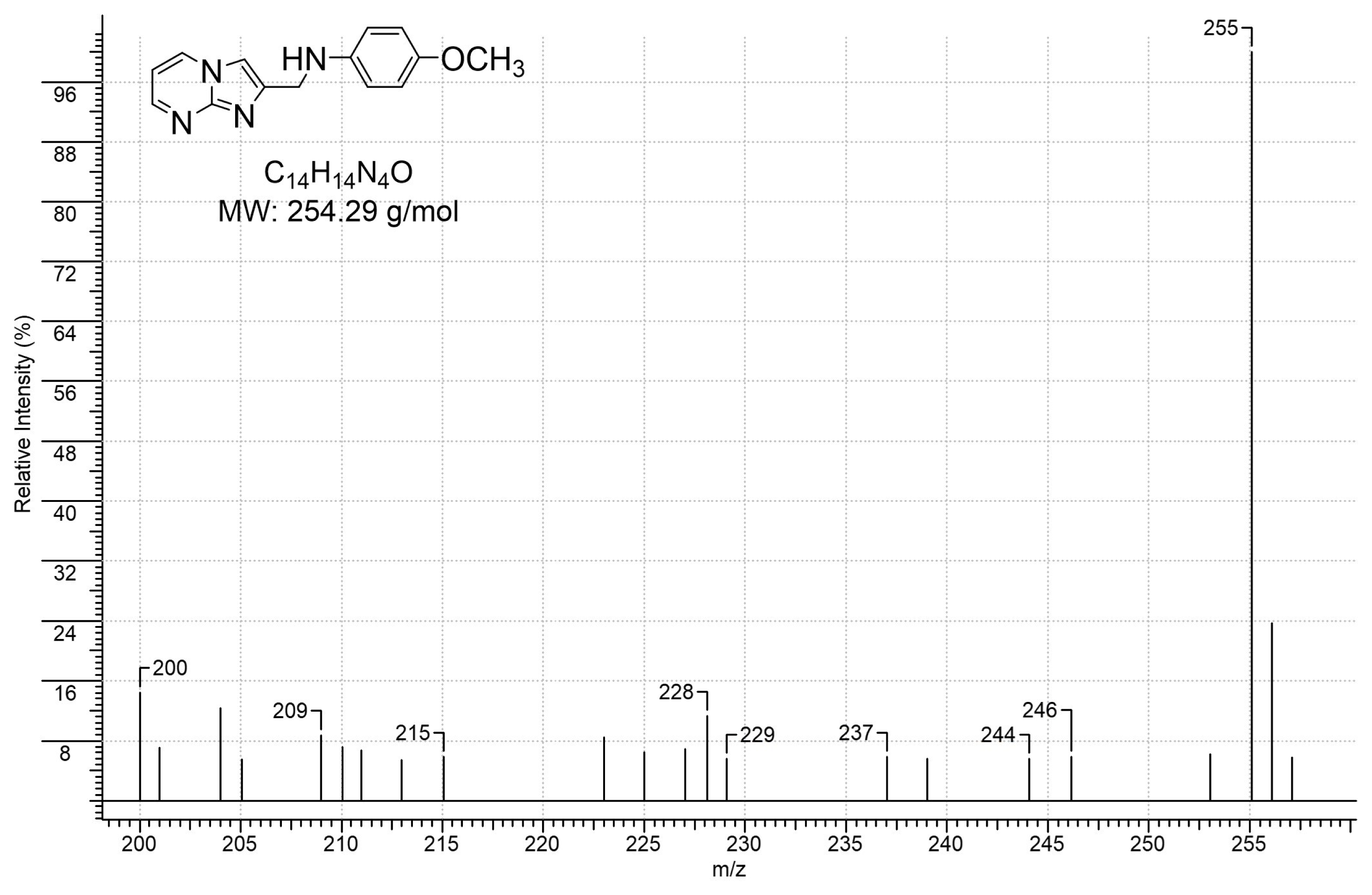

Supplement: Figure S18 — MS spectrum of compound 4c. [file turkjchem-47-5-1064s18.tif]

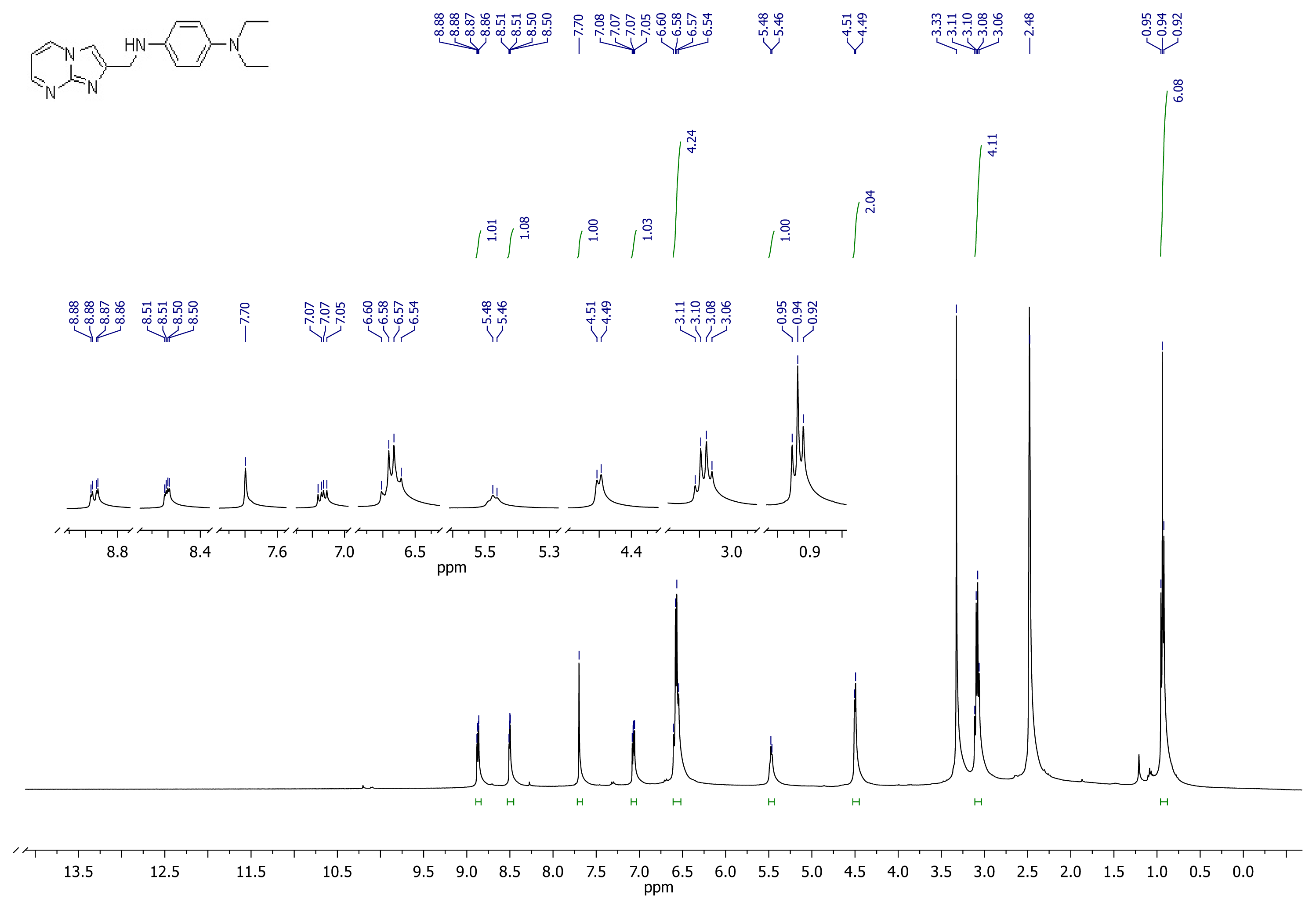

Supplement: Figure S19 — 1H NMR spectrum of compound 4d. [file turkjchem-47-5-1064s19.tif]

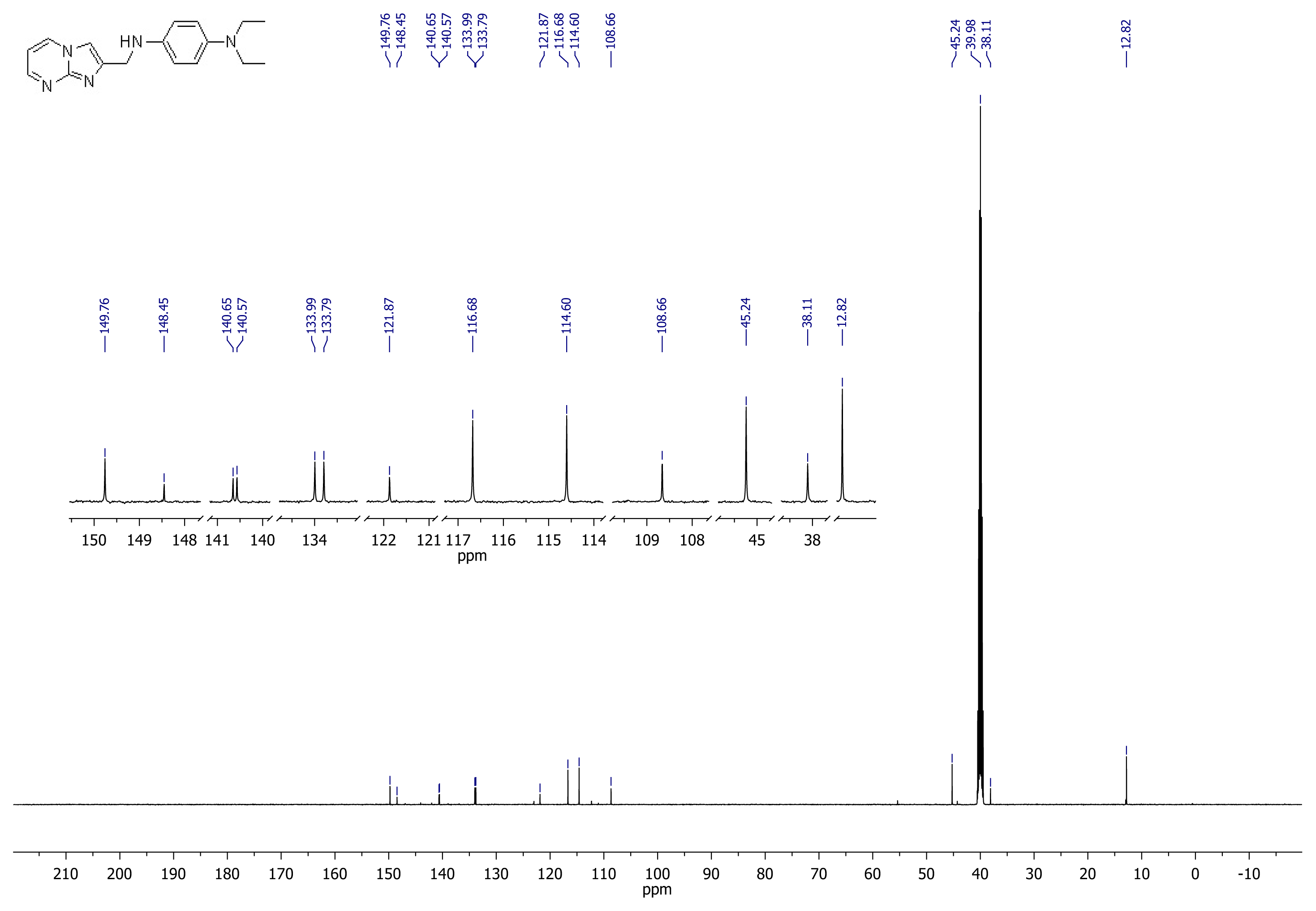

Supplement: Figure S20 — 13C NMR spectrum of compound 4d. [file turkjchem-47-5-1064s20.tif]

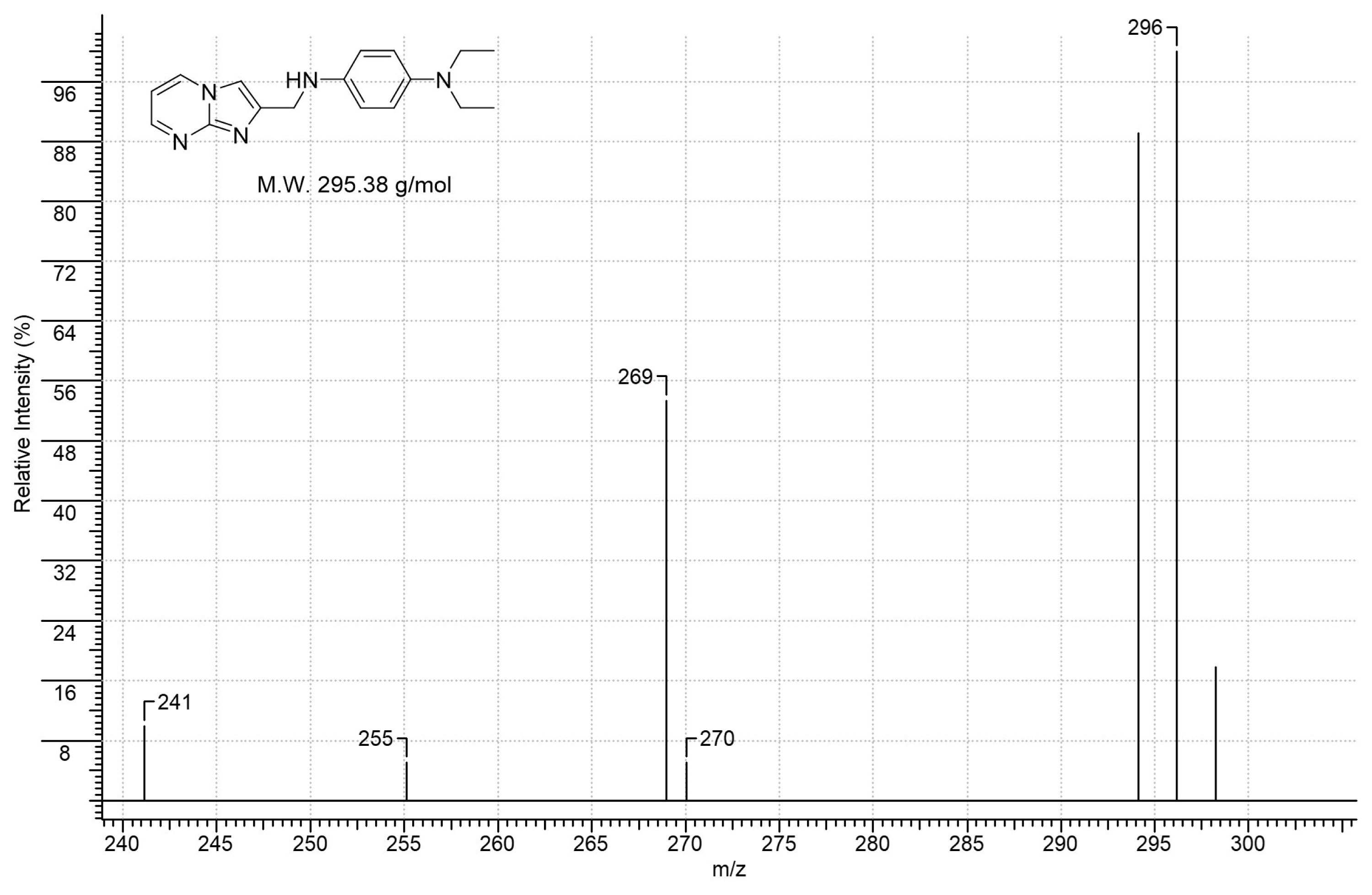

Supplement: Figure S21 — MS spectrum of compound 4d. [file turkjchem-47-5-1064s21.tif]

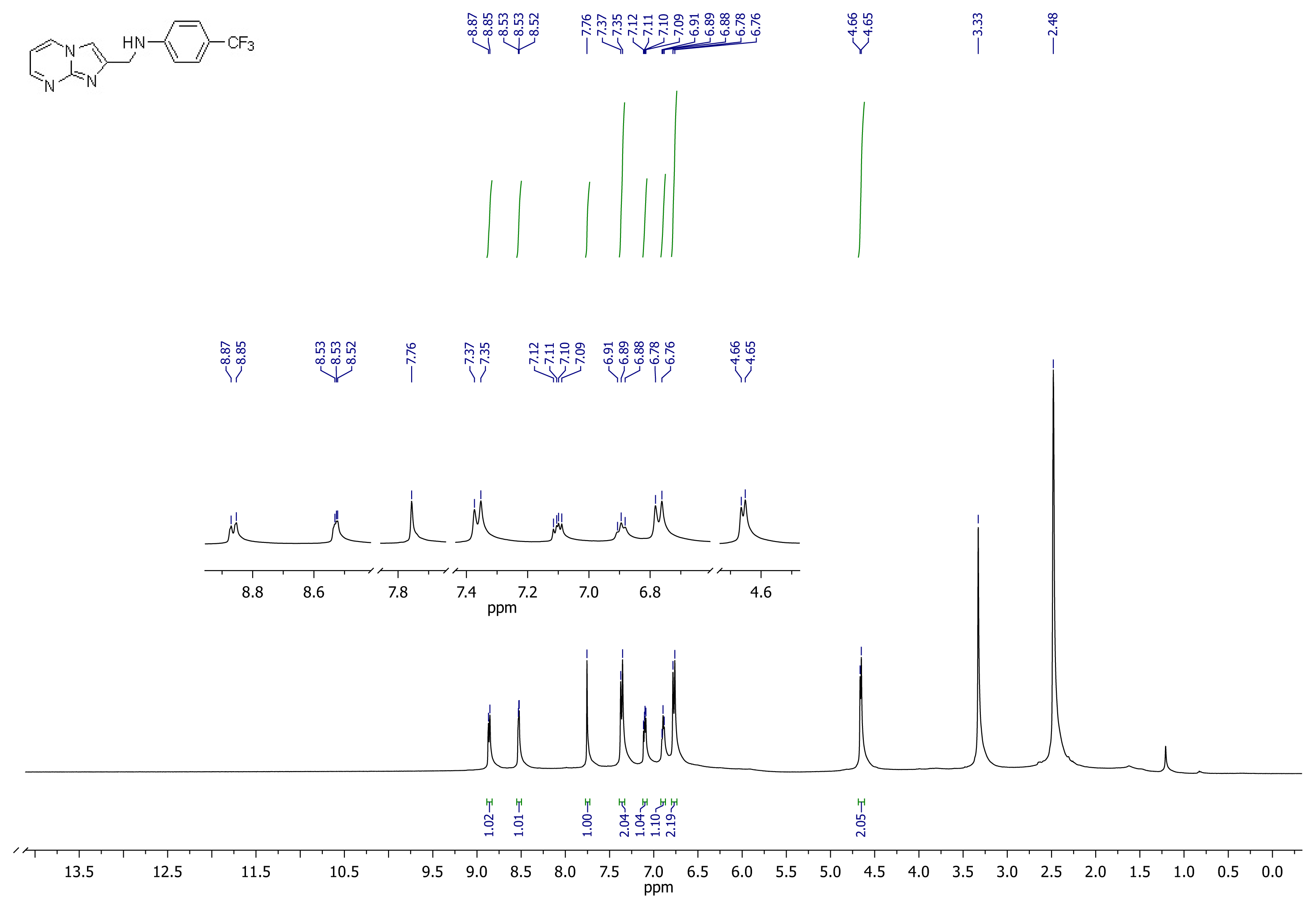

Supplement: Figure S22 — 1H NMR spectrum of compound 4e. [file turkjchem-47-5-1064s22.tif]

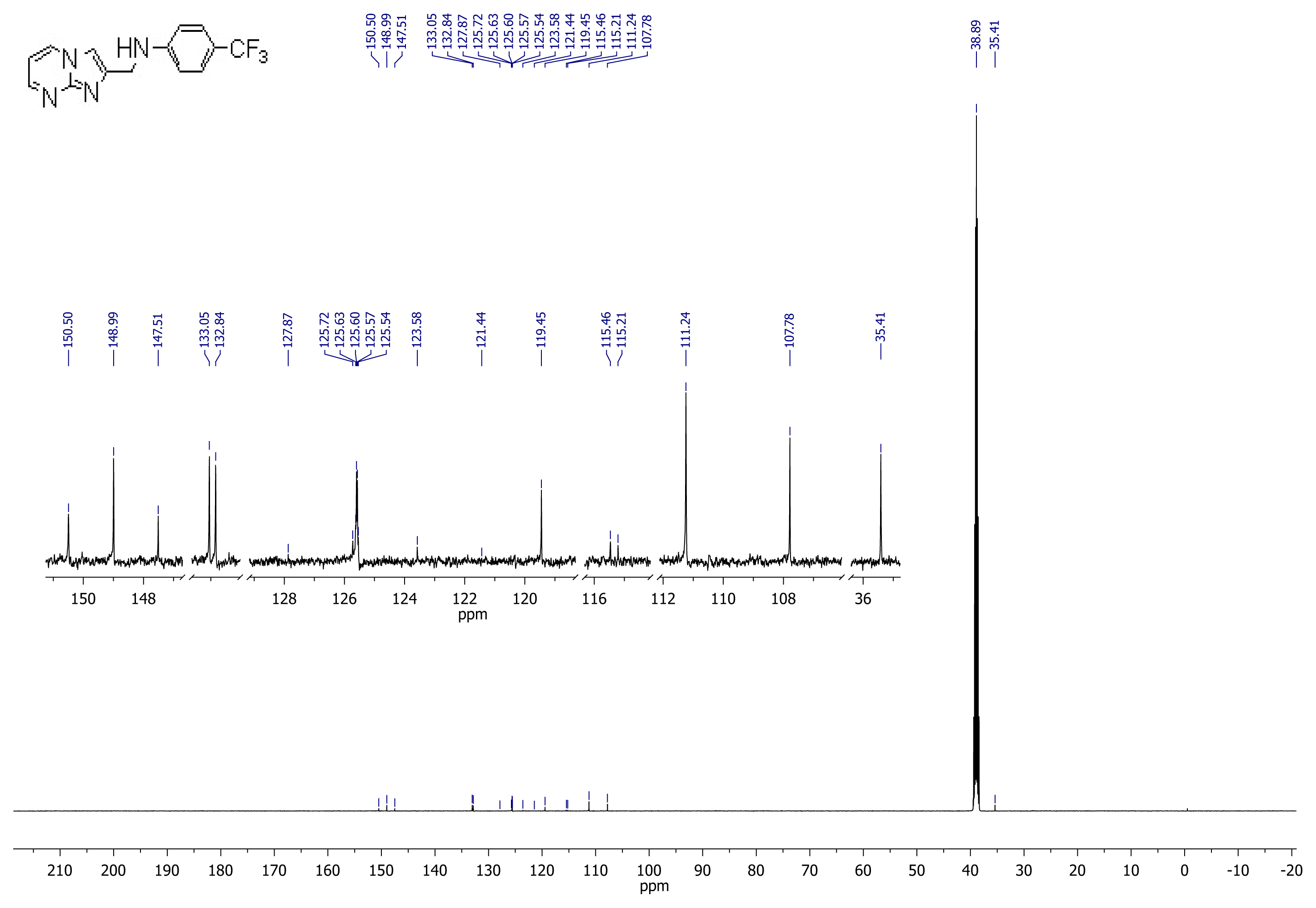

Supplement: Figure S23 — 13C NMR spectrum of compound 4e. [file turkjchem-47-5-1064s23.tif]

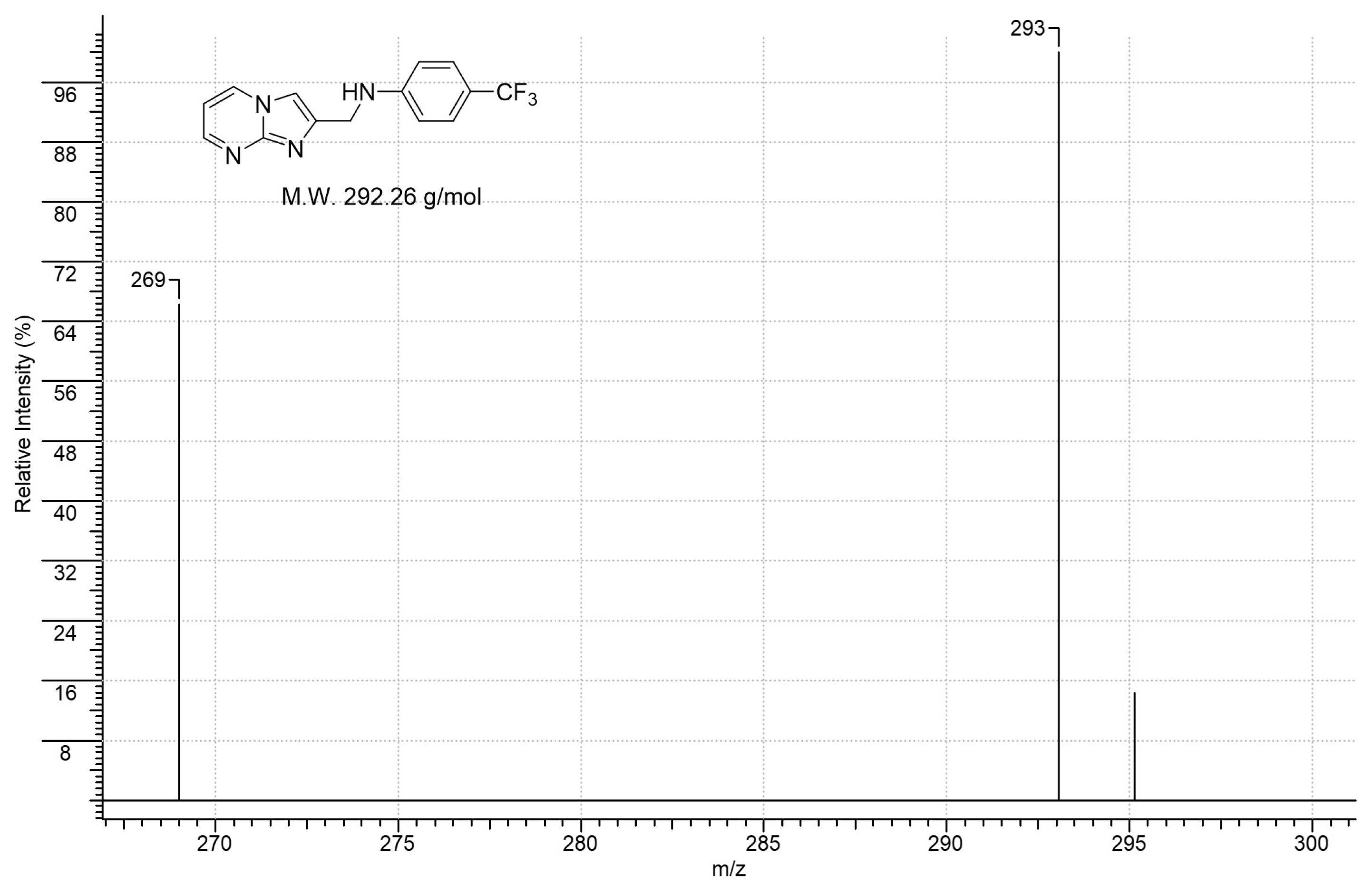

Supplement: Figure S24 — MS spectrum of compound 4e. [file turkjchem-47-5-1064s24.tif]
